# PEGylation of silver nanoparticles by physisorption of cyclic poly(ethylene glycol) for enhanced dispersion stability, antimicrobial activity, and cytotoxicity†

**DOI:** 10.1039/d1na00720c

**Published:** 2021-11-12

**Authors:** Onyinyechukwu Justina Oziri, Yubo Wang, Tomohisa Watanabe, Shuya Uno, Masatoshi Maeki, Manabu Tokeshi, Takuya Isono, Kenji Tajima, Toshifumi Satoh, Shin-ichiro Sato, Yutaka Miura, Takuya Yamamoto

**Affiliations:** Graduate School of Chemical Sciences and Engineering, Hokkaido University Sapporo Hokkaido 060-8628 Japan; Division of Applied Chemistry, Faculty of Engineering, Hokkaido University Sapporo Hokkaido 060-8628 Japan yamamoto.t@eng.hokudai.ac.jp; Laboratory for Chemistry and Life Science, Institute of Innovative Research, Tokyo Institute of Technology 4259 Nagatsutacho, Midori-ku Yokohama Kanagawa 226-8503 Japan

## Abstract

Silver nanoparticles (AgNPs) are practically valuable in biological applications. However, no steady PEGylation has been established, which is essential for internal use in humans or animals. In this study, cyclic PEG (*c*-PEG) without any chemical inhomogeneity is physisorbed onto AgNPs to successfully PEGylate and drastically enhance the dispersion stability against physiological conditions, white light, and high temperature. In contrast, linear HO–PEG–OH and MeO–PEG–OMe do not confer stability to AgNPs, and HS–PEG–OMe, which is often used for gold nanoparticles, sulfidates the surface to considerably degrade the properties. TEM shows an essentially intact nanostructure of *c*-PEG-physisorbed AgNPs even after heating at 95 °C, while complete disturbance is observed for other AgNPs. Molecular weight- and concentration-dependent stabilization by *c*-PEG is investigated, and DLS and *ζ*-potential measurements prove the formation of a *c*-PEG layer on the surface of AgNPs. Furthermore, *c*-PEG-physisorbed AgNPs exhibit persistent antimicrobial activity and cytotoxicity.

## Abstract 2

Cyclic PEG without any chemical inhomogeneity was physiosorbed onto AgNPs to PEGylate and drastically enhance the dispersion stability against a physiological condition, white light, and high temperature, while its linear counterparts could not.

## Graphical abstract

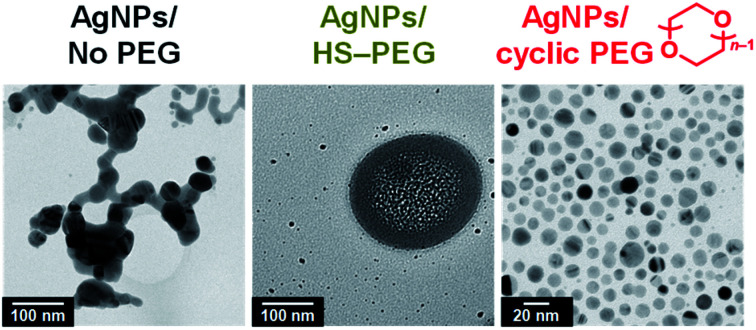

## 1. Introduction

The emerging importance of nanoscience cannot be overemphasized, and the field of nanotechnology has drawn special worldwide attention especially in the cases of noble metals such as gold and silver.^1^ The distinct properties of silver nanoparticles (AgNPs) have led to their broad applications in medical imaging,^2^ drug delivery,^3^ cell electrodes,^4^ biosensors,^5^ cancer diagnosis and treatment,^6^ and cytotoxic agents^7^ as well as antimicrobial agents against a broad spectrum of Gram-negative and Gram-positive bacteria.^8^ The nano-environment of AgNPs has notable effects on their response/activity in many applications. However, unlike gold nanoparticles (AuNPs), AgNPs are not a stable material and susceptible to light, dissolved electrolytes, and various chemical species, and transformations occur leading to aggregation, dissolution, change in structure, activity loss, *etc.*^9^ In diverse fields of nanoscience, the instability of AgNPs often hinders their applications and commercialization. Although several capping agents have been explored for AgNPs, transformations, dissolution and agglomerations in various environments still remain a significant issue.

The use of stabilizers such as cetyltrimethylammonium bromide (CTAB),^10,11^ sodium dodecyl sulfate (SDS),^10,12^ and other surfactants has been well reviewed with limitations of non-biocompatibility and instability. The utmost crucial factors of stability and biocompatibility have been desirable and sought after in the application of nanoparticles. In this regard, poly(ethylene glycol) (PEG), a non-ionic polymer with a flexible structure, is the most commonly used biocompatible polymer accepted by the United States Food and Drug Association and has wide applications including food, commodities, and drugs as well as uses in agriculture and manufacturing industries.^13–16^ In order to use PEG as a stabilizer for metal nanoparticles, especially AuNPs, thiol-functionalized PEG (HS–PEG–OMe) is often employed through the chemisorption between the sulfur atom and metal surface.^17^ However, the use of HS–PEG–OMe for AgNPs results in the formation of a silver sulfide (Ag_2_S) layer on the surface drastically disturbing the nanoparticles' properties^18^ and leading to dissolution,^19,20^ inhibiting PEGylation of AgNPs by the thiol chemisorption. A few reports show that AgNO_3_ is reduced by hydroxy-terminated PEG (HO–PEG–OH), and the resulting nanoparticles are capped by the same polymer in the process.^21–23^ However, the number of reports using this method is quite limited, and thus the capping structure and properties of the formed nanoparticles are not well studied. For these reasons, polyvinylpyrrolidone (PVP) is an alternative polymer often used for AgNPs but has limited biocompatibility compared to PEG.^24–26^ Therefore, stable PEGylation of AgNPs to curb the various restrictions is utmost needed.

**Fig. 1:**
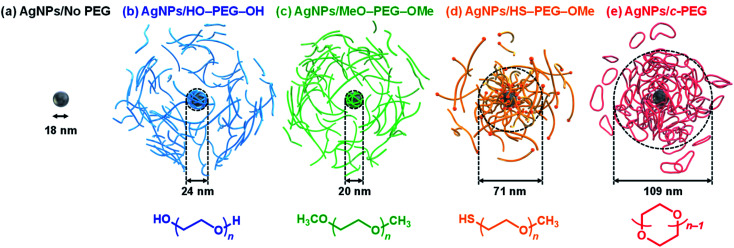
Schematic illustrations of (a) AgNPs/No PEG, (b) AgNPs/HO–PEG–OH, (c) AgNPs/MeO–PEG–OMe, (d) AgNPs/HS–PEG–OMe, and (e) AgNPs/*c*-PEG. The indicated sizes were determined by DLS.

In recent years, attention has been drawn to interesting polymer properties such as melting, diffusion, rheology, crystallization, and phase separation as a result of distinct topological differences.^27,28^ Of the various polymer topologies, cyclic polymers have shown unique physical and chemical properties such as increased glass transition temperature, higher refractive index, less entanglement, slow hydrolytic degradation, and self-assembling behaviors, different from their linear counterparts with the same molecular weight.^29–35^ In biomedical applications, cyclic polymers have promising potential due to their superior properties such as higher efficacy of gene delivery,^36^ higher cancer cell take up,^37^ longer circulation time *in vivo*,^38,39^ and controlled release of drugs.^40^ We reported that cyclic amphiphilic block copolymers form micelles with strong salt and thermal stabilities.^41,42^ Recently, cyclized PEG (*c*-PEG) without any chemical inhomogeneity was found to endow AuNPs with high dispersion stability by physisorption.^43^ Moreover, AuNPs PEGylated by *c*-PEG can be used for the colorimetric detection of bovine serum albumin through a unique complexation.^44^ The strong physisorption of *c*-PEG likely arises from the less entropic penalty upon adsorption to the surface compared to the linear counterparts,^45,46^ which is also suggested by theoretical and computational studies.^47–51^ Bearing this in mind, the physisorption of *c*-PEG is considered suitable for PEGylation and dispersion stabilization of AgNPs because AgNPs are much less chemically stable than AuNPs, and the thiol chemisorption is essentially inapplicable. The present work provides a new approach for the stabilization of AgNPs in the presence of *c*-PEG against physiological conditions, white light, and high temperature, as well as to examine the antimicrobial activity and cytotoxicity of AgNPs. We found that, in contrast to HS–PEG–OMe and other linear PEG, *c*-PEG readily stabilized AgNPs against the various harsh conditions and retained the biological activities (Fig. 1). This result is important as the first stable PEGylation method for AgNPs owing to their potential biomedical applications.

## 2. Experimental

### 2.1 Materials

Citrate-capped AgNPs with a size of 10, 20, and 30 nm (AgNPs_10_, AgNPs_20_, and AgNPs_30_, respectively) dispersed in a 2 mM sodium citrate solution with a silver mass concentration of 0.02 mg mL^−1^ were purchased from nanoComposix, USA and used as received. AgNPs with a size of 80 nm functionalized with methoxy polyethylene glycol sulfhydryl with a molecular weight of 5 kDa (HS–PEG_5k_–OMe) in Milli-Q water with a silver mass concentration of 0.02 mg mL^−1^ were also purchased from nanoComposix, USA and used as received. Poly(ethylene glycol) 2000 (HO–PEG_2k_–OH) (Sigma-Aldrich, Japan), poly(ethylene glycol) 4000 (HO–PEG_3k_–OH) (Kanto Chemical Co., Inc., Japan), and poly(ethylene glycol) 6000 (HO–PEG_9k_–OH) were purified by passing them through a silica gel column using chloroform/methanol (90/10, v/v) as an eluent. *m*PEG-SH, 10k (HS–PEG_9k_–OMe) (Funakoshi Co., Ltd, Japan) was purified by recycling preparative SEC. The AgNPs' and PEGs' names in the parentheses above are the ones used in the paper. The molecular weights of PEG in the catalogs deviated from our measurement to some extent, and the measurement values are used. Tetrahydrofuran (THF), dehydrated and stabilizer free (>99.0%, Kanto Chemicals Co., Japan), anthralin (≥95.0%, Nacalai Co., Japan), silver trifluoroacetate (98%, Sigma-Aldrich, Japan), *n*-heptane (>99.0%, Kanto Chemicals Co., Japan), potassium hydroxide (>99.0%, Kanto Chemicals Co., Japan), iodomethane (>99.0%, Kanto Chemicals Co., Japan), chlorobenzene (>99.0%, Kanto Chemicals Co., Japan), chloroform (>99.0%, Kanto Chemicals Co., Japan), tosyl chloride (>99.0%, Sigma-Aldrich, Japan), acetone (>99.0%, Kanto Chemicals Co., Japan), methanol (>99.6%, Kanto Chemicals Co., Japan), dichloromethane (>99.0%, Kanto Chemicals Co., Japan), magnesium sulfate (>96.0%, Kanto Chemicals Co., Japan), sodium dihydrogen phosphate (>99.0%, FUJIFILM Wako Pure Chemical Co., Japan), disodium hydrogen phosphate (>99.0%, FUJIFILM Wako Pure Chemical Co., Japan), sodium chloride (>99.0%, Kanto Chemicals Co., Japan), chloroform-d (99.6 atom% D, Tokyo Chemical Industry Co., Ltd, Japan), Wakosil C-300 (FUJIFILM Wako Pure Chemical Co., Japan), *E. coli* JM 109 (NIPPON GENE Co., Ltd, Japan), Muller Hinton Broth (Sigma-Aldrich, Japan), ampicillin (Fujita Pharmaceutical. Co., Ltd, Japan), HeLa cells (KAC Co., Ltd, Japan), fetal bovine serum (FBS) (Thermo Fisher Scientific, USA), penicillin–streptomycin, and trypsin (2.5%) (Thermo Fisher Scientific, USA), Dulbecco's modified Eagle's medium (DMEM) with low glucose (Sigma-Aldrich, USA), and D-PBS (−) (FUJIFILM Wako Pure Chemical Co., Japan) were used as received.

### 2.2 Synthesis of *c*-PEG

Cyclization of HO–PEG–OH was carried out by the previously reported method.^52^ Thus, a solution of vacuum dried HO–PEG–OH (5.0 g) and tosyl chloride (190 mg) in 100 mL of dry THF was added over 144 h to a dispersion of powdered potassium hydroxide (3.3 g) in 100 mL of a mixture of dry THF and *n*-heptane (75/25 v/v) at 40 °C using a syringe pump in a dry nitrogen atmosphere. Additional 24 h of stirring was allowed for complete cyclization. The reaction mixture was filtered, and the solvent was removed under reduced pressure. Chloroform was added to the filtrate, washed with brine followed by deionized water. The organic phase was dried with magnesium sulfate, and the solvent was removed under reduced pressure. Silica gel column chromatography was carried out with a mixture of chloroform/acetone (9/1 v/v) to elute polymeric products that underwent intermolecular reactions, followed by a mixture of chloroform/methanol (9/1 v/v) to elute the crude containing *c*-PEG. The crude was dissolved in dichloromethane, and *n*-heptane was slowly added until the solution turned cloudy. The cloudy solution was heated to 40 °C and cooled to 25 °C with two resultant layers. The upper clear layer containing a relatively large proportion of *c*-PEG was collected, and this procedure was repeated several times to obtain pure *c*-PEG as a white solid. The yields of *c*-PEG_2k_, *c*-PEG_3k_, and *c*-PEG_9k_ were 303 (6.1%), 247 (4.9%), and 145 mg (2.9%), respectively. Concerning the very low isolated yields, the purity was prioritized over the yield, resulting in a major loss during the isolation.

### 2.3 Synthesis of MeO–PEG–OMe

Methylation of HO–PEG–OH was carried out according to the previous method.^52^ Typically, in a dry nitrogen atmosphere, chlorobenzene (20 mL) was added to finely powdered potassium hydroxide (2.3 g), and the mixture was stirred at 25 °C. Iodomethane (0.35 g) was added to the mixture, followed by slow addition of HO–PEG_2k_–OH (5.0 g) dissolved in chlorobenzene (50 mL) over 25 min. The mixture was stirred for 24 h. Filtration was carried out, and the filtrate was reduced to a small volume under reduced pressure, followed by washing with distilled water and deionized water. The organic phase was dried with magnesium sulfate and concentrated under reduced pressure. The residue was applied to a silica gel column with a mixture of chloroform/acetone (9/1 v/v), and the product was eluted with a mixture of chloroform/methanol (9/1 v/v). The solvent was removed and vacuum dried to obtain dimethylated poly(ethylene glycol) (MeO–PEG_2k_–OMe) (3.6 g, 72%) as a white solid.

### 2.4 NMR

^1^H NMR (400 MHz) and ^13^C NMR (100 MHz) were recorded on a JEOL JNM-ESC400 instrument at room temperature at a polymer concentration of 20 mg mL^−1^. Deuterated chloroform was used as a solvent.

### 2.5 SEC

Size exclusion chromatography measurements were performed on a Shodex GPC-101 gel permeation chromatography system (Shodex DU-2130 dual pump, Shodex RI-71 reflective index detector, and Shodex ERC-3125SN degasser) equipped with a Shodex KF-G guard column (4.6 mm × 10 mm; pore size, 8 μm) and two Shodex KF-804L columns (8 mm × 300 mm) in series. THF was used as an eluent at a flow rate of 1.0 mL min^−1^. Calibration was performed with PEG standard samples.

### 2.6 MALDI-TOF MS

Matrix-assisted laser desorption/ionization time-of-flight mass spectrometry (MALDI-TOF MS) was performed at the Open Facility, Hokkaido University using an ABSCIEX TOF/TOF 5800 mass spectrometer. PEG (1.5 mg) dissolved in THF (10 μL) was mixed with a matrix (anthralin, 40 mg mL^−1^, 25 μL) and an ionization agent (silver trifluoroacetate, 40 mg mL^−1^, 10 μL). The mixture (0.4 μL) was dropcast on an opti-TOF 384-Well Insert (123 × 81 mm) plate for the measurement.

### 2.7 Recycling preparative SEC

A Japan Analytical Industry LC-908 recycling preparative HPLC system (Hitachi L-7110 pump and JAI RI detector RI-5) was used. JAIGEL-2H and 3H columns and a pre-column were connected in series. Chloroform was used as a solvent, and the flow rate was set at 3.5 mL min^−1^.

### 2.8 UV-Vis spectroscopy

UV-Vis absorption spectra were recorded using a JASCO Ubset V-670 spectrophotometer at 25 °C in a micro quartz cuvette (M25-UV2, GL Science Inc., Japan) with a path length of 10 mm. Deionized water was used as a blank. Spectra were acquired at a wavelength range of 300–800 nm. Optical density at 600 nm (OD_600_) in the antimicrobial activity experiment was determined by the intensity of an incubated specimen at 600 nm with that of the medium subtracted.

### 2.9 DLS and *ζ*-potential

DLS and *ζ*-potential measurements were carried out using a Zetasizer Nano ZS instrument (He–Ne laser, 633 nm, max 4 mW, Malvern Panalytical Ltd). A micro quartz cuvette (ZEN2112, Hellma Analytics) and Zetasizer nano cell (DTS1060, Malvern Instruments, Ltd) were used. Measurements were carried out at 25 °C with a 120 s equilibration time. A cumulant analysis performed using the inbuilt software of the instrument was used to determine the *z*-average size.

### 2.10 Preparation of AgNPs/HO–PEG–OH, AgNPs/MeO–PEG–OMe, AgNPs/HS–PEG–OMe, and AgNPs/*c*-PEG

Typically, an aqueous dispersion of AgNPs_10_ (0.54 mL) was added to HO–PEG_9k_–OH, MeO–PEG_9k_–OMe, HS–PEG_9k_–OMe, or *c*-PEG_9k_ (1.5 mg) in a 1.5 mL Eppendorf tube, and the mixture was vortexed for 1 min to form AgNPs_10_/HO–PEG_9k_–OH, AgNPs_10_/MeO–PEG_9k_–OMe, AgNPs_10_/HS–PEG_9k_–OMe, or AgNPs_10_/*c*-PEG_9k_, respectively. The PEG concentration was varied by changing the amount of PEG. AgNPs_10_/No PEG was prepared by vortex mixing an aqueous dispersion of AgNPs_10_ (0.54 mL).

### 2.11 Stability in a PBS buffer

A tenfold-concentrated phosphate-buffered saline (PBS) solution (pH 7.4, NaCl 1500 mM, Na_2_HPO_4_ 81 mM, NaH_2_PO_4_ 14.7 mM) was prepared in advance. AgNPs_10_/No PEG, AgNPs_10_/HO–PEG_9k_–OH, AgNPs_10_/MeO–PEG_9k_–OMe, AgNPs_10_/HS–PEG_9k_–OMe, or AgNPs_10_/*c*-PEG_9k_ (0.54 mL) prepared above was placed in a micro quartz cuvette. Subsequently, the tenfold-concentrated PBS solution (0.06 mL) was added to the cuvette, and the resulting mixture was 0.6 mL with pH 7.4 and a NaCl concentration of 150 mM with a PEG concentration of 0.25 wt%. A time-course UV-Vis measurement was performed for 1000 min.

For ESI Movie 1,† an aqueous dispersion of AgNPs_10_ (0.54 mL) was added to HO–PEG_9k_–OH, MeO–PEG_9k_–OMe, HS–PEG_9k_–OMe, or *c*-PEG_9k_ (1.5 mg) in a glass vial and mixed by pipetting to dissolve PEG to form AgNPs_10_/HO–PEG_9k_–OH, AgNPs_10_/MeO–PEG_9k_–OMe, AgNPs_10_/HS–PEG_9k_–OMe, or AgNPs_10_/*c*-PEG_9k_, respectively. AgNPs_10_/No PEG was prepared by pipetting an aqueous dispersion of AgNPs_10_ (0.54 mL). Subsequently, the tenfold-concentrated PBS solution (0.06 mL) was added to the glass vial and mixed by pipetting. The resulting mixture was 0.6 mL with pH 7.4 and a NaCl concentration of 150 mM with a PEG concentration of 0.25 wt%. A color change was observed.

### 2.12 *c*-PEG's molecular weight-dependent stability

An aqueous dispersion of AgNPs_10_ (0.54 mL) was added to *c*-PEG_2k_, *c*-PEG_3k_, or *c*-PEG_9k_ (0.15 mg) in a 1.5 mL Eppendorf tube, and the mixture was vortexed for 1 min to form AgNPs_10_/*c*-PEG_2k_, AgNPs_10_/*c*-PEG_3k_, or AgNPs_10_/*c*-PEG_9k_, respectively. Subsequently, the tenfold-concentrated PBS solution (0.06 mL) was added to the cuvette, and the resulting mixture was 0.6 mL with pH 7.4 and a NaCl concentration of 150 mM with a PEG concentration of 0.25 wt%. A time-course UV-Vis measurement was performed for 1000 min.

### 2.13 *c*-PEG's concentration-dependent stability

An aqueous dispersion of AgNPs_10_ (0.54 mL) was added to *c*-PEG_9k_ (0.3, 1.5, 3.0, or 7.5 mg) in a 1.5 mL Eppendorf tube, the mixture was vortexed for 1 min to form AgNPs_10_/*c*-PEG_9k_. Subsequently, the tenfold-concentrated PBS solution (0.06 mL) was added to the cuvette, and the resulting mixture was 0.6 mL with pH 7.4 and a NaCl concentration of 150 mM with a PEG concentration of 0.05, 0.25, 0.40, or 1.25 wt%, respectively. A time-course UV-Vis measurement was performed for 1000 min.

### 2.14 AgNPs' size-dependent stability

*c*-PEG_9k_ (1.5 mg) was added to an aqueous dispersion of AgNPs_10_, AgNPs_20_, or AgNPs_30_ (0.54 mL), and the mixture was vortexed for 1 min. Subsequently, the tenfold-concentrated PBS solution (0.06 mL) was added to the cuvette, and the resulting mixture was 0.6 mL with pH 7.4 and a NaCl concentration of 150 mM with a PEG concentration of 0.25 wt%. A time-course UV-Vis measurement was performed for 1000 min.

### 2.15 Stability in CaCl_2_ solution

A tenfold-concentrated calcium chloride solution (CaCl_2_ 100 mM, pH unadjusted) was prepared in advance. AgNPs_10_/No PEG, AgNPs_10_/HO–PEG_9k_–OH, AgNPs_10_/MeO–PEG_9k_–OMe, AgNPs_10_/HS–PEG_9k_–OMe, or AgNPs_10_/*c*-PEG_9k_ (0.54 mL) prepared above was placed in a micro quartz cuvette. Subsequently, the tenfold-concentrated CaCl_2_ solution (0.06 mL) was added to the cuvette, and the resulting mixture was 0.6 mL with 10 mM of CaCl_2_ with a PEG concentration of 0.25 wt%. A time-course UV-Vis measurement was performed for 1000 min.

### 2.16 Stability against white light

An aqueous dispersion of AgNPs_10_ (2.1 mL) was two times diluted with deionized water (2.1 mL) and added to HO–PEG_9k_–OH, MeO–PEG_9k_–OMe, HS–PEG_9k_–OMe, or *c*-PEG_9k_ (10.5 mg) in a 50 mL Falcon tube. The mixture was vortexed for 1 min to form AgNPs_10_/HO–PEG_9k_–OH, AgNPs_10_/MeO–PEG_9k_–OMe, AgNPs_10_/HS–PEG_9k_–OMe, or AgNPs_10_/*c*-PEG_9k_, respectively, where the PEG concentration was 0.25 wt%. AgNPs_10_/No PEG was prepared by diluting AgNPs (2.1 mL) with deionized water (2.1 mL) and vortexed for 1 min. The mixtures were kept under 860–990 lux light intensity using a white light emitting tube at 25 °C for 35 d. 0.60 mL of the mixtures was withdrawn from the Falcon tubes immediately after mixing (day 0) and subsequently at a 7 day interval for an absorption measurement. The measured samples were not returned to the Falcon tubes.

### 2.17 Stability against various temperatures

An aqueous dispersion of AgNPs_10_ (0.30 mL) was two times diluted with deionized water (0.30 mL) and added to HO–PEG_9k_–OH, MeO–PEG_9k_–OMe, HS–PEG_9k_–OMe, or *c*-PEG_9k_ (1.5 mg) in a 1.5 mL Eppendorf tube. The mixture was vortexed for 1 min to form AgNPs_10_/HO–PEG_9k_–OH, AgNPs_10_/MeO–PEG_9k_–OMe, AgNPs_10_/HS–PEG_9k_–OMe, or AgNPs_10_/*c*-PEG_9k_, respectively, where the PEG concentration was 0.25 wt%. AgNPs_10_/No PEG was prepared by diluting AgNPs (0.30 mL) with deionized water (0.30 mL) and vortexed for 1 min. The mixtures were incubated for 4 h at 4, 37, or 95 °C, and UV-Vis absorption spectra were recorded.

### 2.18 TEM

A few drops from the above AgNPs_10_/No PEG, AgNPs_10_/HS–PEG_9k_–OMe, or AgNPs_10_/*c*-PEG_9k_ heated at 95 °C for 4 h were placed on a carbon coated Formvar TEM grid and air blown with a blower. Measurements were performed with a Japan Electron Optics Laboratory JEM-2010 operated at 200 kV.

### 2.19 Antimicrobial activity

*E. coli* was grown in a Muller Hinton Broth (MHB) medium containing ampicillin (100 μg mL^−1^) at 37 °C for 24 h and standardized using 0.5 McFarland standard (10^8^ CFU mL^−1^). A tenfold-concentrated PBS solution (300 μL, pH 7.4, NaCl 1500 mM) was added to AgNPs_10_/No PEG, AgNPs_10_/HO–PEG_9k_–OH, AgNPs_10_/MeO–PEG_9k_–OMe, AgNPs_10_/HS–PEG_9k_–OMe, or AgNPs_10_/*c*-PEG_9k_ (2.7 mL) with a PEG concentration of 0.25 wt% and incubated for 24 h. The resulting mixture was centrifuged at 3000 rpm for 20 min, and the supernatant or dispersion was ultrafiltered to reduce the volume to 100 μL and mixed with 10 μL of 10^5^ CFU mL^−1^*E. coli* in a 1.5 mL Eppendorf tube. The mixture was added to a test tube containing an MHB medium (2.9 mL) and incubated at 37 °C and 200 rpm for 24 h. UV-Vis absorption spectra were recorded.

### 2.20 Cytotoxicity

HO–PEG_9k_–OH, MeO–PEG_9k_–OMe, HS–PEG_9k_–OMe, or *c*-PEG_9k_ was dissolved into DMEM (FBS (−)) at a concentration of 5 mg mL^−1^. The PEG solution was mixed with an aqueous dispersion of AgNPs (20 μg mL^−1^) at a 1 : 1 volume ratio. The mixture was diluted with DMEM (FBS (−)) by 10-fold for the final concentrations of AgNPs (1.0 μg mL^−1^) and of PEG (0.25 mg mL^−1^). For AgNPs/No PEG, AgNPs (20 μg mL^−1^) were diluted with DMEM (FBS (−)) to form AgNPs (1.0 μg mL^−1^). HeLa cells were cultured in cell culture dishes (Eppendorf) containing DMEM with 10% FBS, 100 U mL^−1^ penicillin, and 100 μg mL^−1^ streptomycin at 37 °C in 5% CO_2_. For the cell viability assay, the cells were seeded at a concentration of 6 × 10^3^ per well in a 96 well microplate (Thermo Scientific) and grown for 24 h. The cells were treated with 100 μL of AgNPs_10_/No PEG, AgNPs_10_/HO–PEG_9k_–OH, AgNPs_10_/MeO–PEG_9k_–OMe, AgNPs_10_/HS–PEG_9k_–OMe, or AgNPs_10_/*c*-PEG_9k_ followed by incubation for 2 h at 37 °C in 5% CO_2_. After the incubation, the cells were washed with D-PBS (−), and the medium was replaced with 100 μL of fresh DMEM (FBS (+)). The cells were additionally incubated for 24 h at 37 °C in 5% CO_2_. The cell viability was measured using a CellTiter-Glo 2.0 cell viability assay kit (Promega) according to the manufacturer's protocol.

### 2.21 Cell scratch assay

AgNPs_10_/No PEG, AgNPs_10_/HO–PEG_9k_–OH, AgNPs_10_/MeO–PEG_9k_–OMe, AgNPs_10_/HS–PEG_9k_–OMe, and AgNPs_10_/*c*-PEG_9k_ were prepared in the same manner as the cell viability assay with the final concentrations of AgNPs (1.0 μg mL^−1^) and of PEG (0.25 mg mL^−1^) in DMEM (FBS (−)). HeLa cells were seeded at a concentration of 8 × 10^4^ per well in a 24 well microplate (Corning) and grown for 24 h. The cells were treated with 250 μL of AgNPs_10_/No PEG, AgNPs_10_/HO–PEG_9k_–OH, AgNPs_10_/MeO–PEG_9k_–OMe, AgNPs_10_/HS–PEG_9k_–OMe, or AgNPs_10_/*c*-PEG_9k_ followed by incubation for 2 h at 37 °C in 5% CO_2_. After incubation, the cells were washed with D-PBS (−) and scratched using a pipette tip followed by washing with D-PBS (−). The medium was replaced with 100 μL of fresh DMEM (FBS (+)). The scratched regions were observed using a microscope (BZ-X800, Keyence). Subsequently, the cells were incubated for 22 h at 37 °C in 5% CO_2_, and the scratched regions were measured again.

## 3. Results and discussion

### 3.1 Synthesis of *c*-PEG and MeO–PEG–OMe

HO–PEG–OH with a molecular weight of 2, 3, and 9 kDa (HO–PEG_2k_–OH, HO–PEG_3k_–OH, and HO–PEG_9k_–OH, respectively) was successfully cyclized by etherification. Thus, the chain ends of HO–PEG–OH were intramolecularly connected in the presence of tosyl chloride and potassium hydroxide in dilution. Highly pure *c*-PEG_2k_, *c*-PEG_3k_, and *c*-PEG_9k_ were obtained after column chromatography and repeated separation using dichloromethane and *n*-heptane. SEC of *c*-PEG showed a unimodal trace with a peak shift to the lower molecular weight region compared to the prepolymer HO–PEG–OH (Fig. S1†). The decrease in the hydrodynamic volume resulting from cyclization caused the shift in the apparent molecular weight. For example, *M*_p,SEC_ decreased from 9640 for HO–PEG_9k_–OH to 6040 for *c*-PEG_9k_ (Table 1 and Fig. S1c†). ^13^C NMR spectra showed a complete disappearance of the peaks at 61.8 and 72.5 ppm from the carbon atoms adjacent to the hydroxyl end groups of HO–PEG–OH, thus confirming the elimination of the chain ends (Fig. S2†). ^1^H NMR of *c*-PEG also gave a single peak unlike that of HO–PEG–OH, which showed the distinguishable signals from the methylene protons close to the chain ends (Fig. S3†). MALDI-TOF mass spectrometry of *c*-PEG and HO–PEG–OH further gave a striking difference in their isotopic distributions (Fig. S4†). For example, HO–PEG_2k_–OH gave a peak at *m*/*z* = 2062.13 for [HO(C_2_H_4_O)_44_H + Ag]^+^, where Ag^+^ was from silver trifluoroacetate, an ionization agent, whereas *c*-PEG_2k_ had a peak at *m*/*z* = 2044.26 for [(C_2_H_4_O)_44_ + Ag]^+^ with the difference arising from the elimination of a water molecule. However, a MALDI-TOF mass spectrum for PEG_9k_ was not obtainable due to its large molecular weight. The expected diameter of *c*-PEG_2k_, *c*-PEG_3k_, and *c*-PEG_9k_ was 4.5, 6.8, and 22 nm, respectively, when they form an ideal right circular conformation. Furthermore, methylation of HO–PEG–OH resulted in the successful synthesis of MeO–PEG–OMe with features similar to its precursor HO–PEG–OH in terms of the appearance of the carbon atoms adjacent to the methoxy end group (Fig. S2†). UV-Vis spectroscopy showed no absorbance from any of these PEGs at 300–800 nm wavelength (Fig. S5†), suggesting that the PEG samples are free of impurity and suitable for optical investigations of AgNPs.

**Table 1:** Properties of PEG by SEC

	*M*_n,SEC_ a (g mol^−1^)	*M*_p,SEC_ a (g mol^−1^)	*M*_w_/*M*_n_	Ideal diameter of *c*-PEG (nm)
HO–PEG_2k_–OH	1740	1760	1.05	—
MeO–PEG_2k_–OMe	1780	1760	1.05	—
HS–PEG_2k_–OMe	1690	1760	1.05	—
*c*-PEG_2k_	1170	1170	1.04	4.5
HO–PEG_3k_–OH	2640	2690	1.05	—
MeO–PEG_3k_–OMe	2660	2690	1.04	—
*c*-PEG_3k_	1750	1760	1.05	6.8
HO–PEG_9k_–OH	8690	9640	1.05	—
MeO–PEG_9k_–OMe	8330	8770	1.05	—
HS–PEG_9k_–OMe	9480	9640	1.04	—
*c*-PEG_9k_	5190	6040	1.06	22

a Determined by SEC in THF using PEG standards.

### 3.2 Physisorption of *c*-PEG to AgNPs

**Fig. 2:**
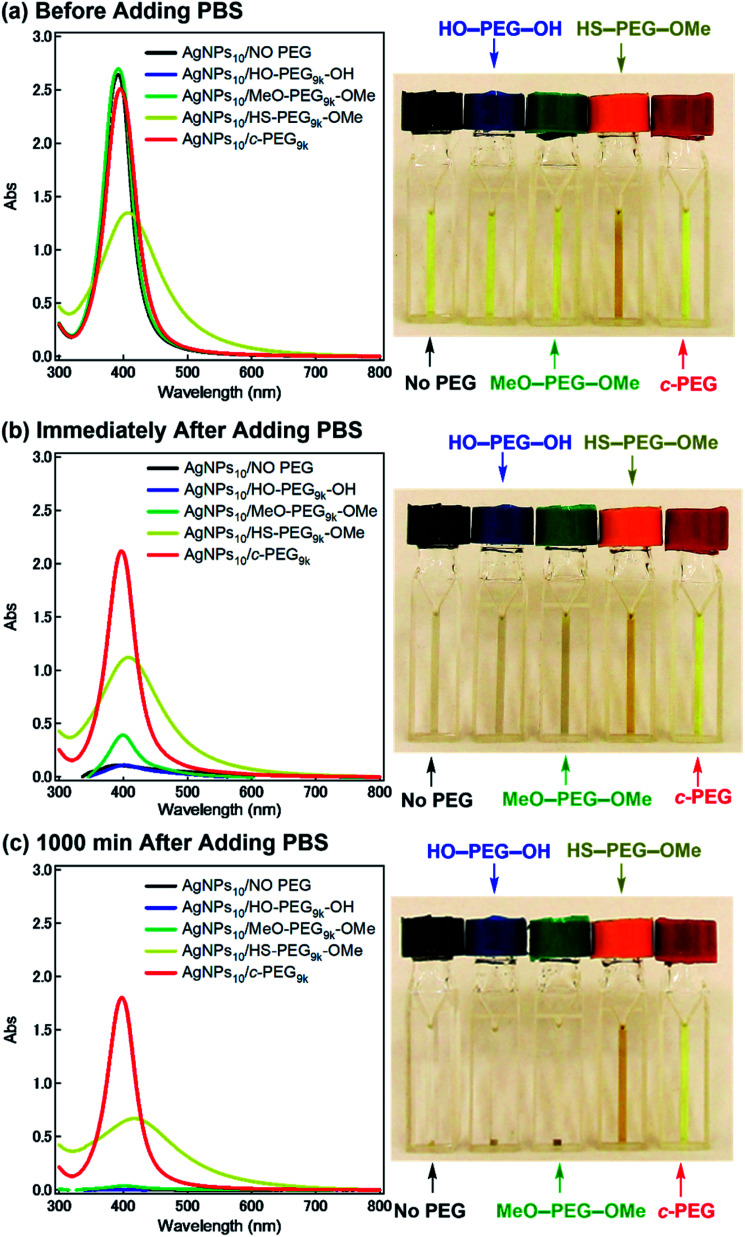
Stability test of AgNPs/PEG against physiological conditions (PBS with pH 7.4 and a NaCl concentration of 150 mM). UV-Vis spectra and photographs of AgNPs_10_/No PEG (black), AgNPs_10_/HO–PEG_9k_–OH (blue), AgNPs_10_/MeO–PEG_9k_–OMe (green), AgNPs_10_/HS–PEG_9k_–OMe (yellow/orange), and AgNPs_10_/*c*-PEG_9k_ (red) with a PEG concentration of 0.25 wt% (a) before (b) immediately after and (c) 1000 min after the addition of a concentrated PBS solution. The resulting dispersions were pH 7.4 and 150 mM of NaCl.

According to the procedure we previously established,^43^ HO–PEG–OH, MeO–PEG–OMe, HS–PEG–OMe, or *c*-PEG with a molecular weight of 2, 3, or 9 kDa (PEG_2k_, PEG_3k_, or PEG_9k_, respectively), was simply mixed with an aqueous dispersion of AgNPs with a size of 10, 20, or 30 nm (AgNPs_10_, AgNPs_20_, or AgNPs_30_, respectively). The addition of HO–PEG–OH, MeO–PEG–OMe, or *c*-PEG to AgNPs had no effect on the surface plasmon resonance (SPR) shown in Fig. 2a. The UV-Vis absorption spectra and visual color were almost identical to those of the AgNPs dispersion without PEG, and *λ*_max_ remained at 398 nm. On the other hand, the addition of HS–PEG_9k_–OMe abruptly reduced the absorption, broadened the peak, and deepened the yellow color of the AgNPs dispersion to yellowish brown. This change in SPR is explained as being a result of increase in the local dielectric environment upon thiol coordination to the Ag surface.^19^

DLS and *ζ*-potential measurements proved the distinct formation of a *c*-PEG layer on the surface of AgNPs as in the case of AuNPs.^43^ Thus, by DLS, AgNPs_10_/No PEG had a size of 18 nm, which on addition of HO–PEG_9k_–OH and MeO–PEG_9k_–OMe slightly enlarged to 24 and 20 nm, respectively (Fig. 1 and Table 2). On the other hand, a significant increase was seen for AgNPs_10_/HS–PEG_9k_–OMe with 71 nm and AgNPs_10_/*c*-PEG_9k_ with 109 nm. Moreover, an increase in the size with increase in the molecular weight of *c*-PEG was evident. In the case of AgNPs_10_, complexation with *c*-PEG_2k_, *c*-PEG_3k_, and *c*-PEG_9k_ resulted in 35, 77, and 109 nm in size, respectively. This molecular weight dependence is consistent with the previously reported adsorption of cyclic PEG and cyclic poly(dimethylsiloxane) on silica.^45,46^ When AgNPs_20_ and AgNPs_30_ were used with *c*-PEG_9k_ (73 and 71 nm, respectively), the increase in size was less intense compared to AgNPs_10_ (109 nm). Thus, *c*-PEG_9k_ with a diameter of 22 nm in the ideal right circular conformation exhibited the strongest interaction with AgNPs_10_ with a size of 18 nm.

**Table 2:** DLS size of AgNPs/No PEG, AgNPs/HO–PEG–OH, AgNPs/MeO–PEG–OMe, AgNPs/HS–PEG–OMe, and AgNPs/*c*-PEG with various AgNPs' sizes and PEG's molecular weights^a^

	AgNPs_10_	AgNPs_20_	AgNPs_30_
No PEG	18	26	35
HO–PEG_2k_–OH	19	30	36
HO–PEG_3k_–OH	23	35	41
HO–PEG_9k_–OH	24	33	38
MeO–PEG_2k_–OMe	32	36	40
MeO–PEG_3k_–OMe	32	34	38
MeO–PEG_9k_–OMe	20	32	41
HS–PEG_2k_–OMe	43	51	49
HS–PEG_9k_–OMe	71	59	59
*c*-PEG_2k_	35	35	45
*c*-PEG_3k_	77	45	49
*c*-PEG_9k_	109	73	71

a Units are in nm.

Due to citrate anions existing at the surface of AgNPs, the *ζ*-potential of AgNPs_10_/No PEG showed a negative value of −31 mV, which on addition of non-ionic PEG reduced to −25 mV for AgNPs_10_/HO–PEG_9k_–OH and −16 mV for AgNPs_10_/MeO–PEG_9k_–OMe (Table 3). A neutral PEG layer on the surface was reported to shield the negative charges of citrate thus decreasing the magnitude of the *ζ*-potential.^53^ A near zero potential was seen for AgNPs_10_/HS–PEG_9k_–OMe and AgNPs_10_/*c*-PEG_9k_ with −2 mV. This suggests that chemisorption of HS–PEG_9k_–OMe and physisorption of *c*-PEG to the surface of AgNPs shield the charge more efficiently. A significant decrease in magnitude of the *ζ*-potential by the addition of *c*-PEG was also previously observed in AuNPs.^43^ Furthermore, an increase in molecular weight led to a reduction in the *ζ*-potential: AgNPs_10_/*c*-PEG_2k_ gave −12 mV, AgNPs_10_/*c*-PEG_3k_ was −6 mV, and a further reduction to −2 mV in AgNPs_10_/*c*-PEG_9k_. Thus, a thicker layer formed by *c*-PEG with a higher molecular weight shielded the charge more effectively.

**Table 3:** *ζ*-Potential of AgNPs/No PEG, AgNPs/HO–PEG–OH, AgNPs/MeO–PEG–OMe, AgNPs/HS–PEG–OMe, and AgNPs/*c*-PEG with various AgNPs' sizes and PEG's molecular weights^a^

	AgNPs_10_	AgNPs_20_	AgNPs_30_
No PEG	−31	−33	−40
HO–PEG_2k_–OH	−23	−27	−31
HO–PEG_3k_–OH	−24	−24	−26
HO–PEG_9k_–OH	−25	−29	−33
MeO–PEG_2k_–OMe	−26	−34	−35
MeO–PEG_3k_–OMe	−28	−36	−33
MeO–PEG_9k_–OMe	−16	−20	−22
HS–PEG_2k_–OMe	−18	−17	−18
HS–PEG_9k_–OMe	−2	−1	−1
*c*-PEG_2k_	−12	−13	−15
*c*-PEG_3k_	−6	−10	−9
*c*-PEG_9k_	−2	−2	−2

a Units are in mV.

The adsorption of *c*-PEG on AgNPs is probably an enthalpically favorable and entropically unfavorable process. Because the number of conformations of *c*-PEG in the unadsorbed state is limited compared to that of HO–PEG–OH and MeO–PEG–OMe, the entropic loss upon the adsorption of *c*-PEG is expected to be smaller.^45–51^ On the other hand, the adsorption enthalpy would be similar for both *c*-PEG and the linear counterparts because they have the same chemical structure of the repeating units and the same molecular weight. Based on this, the total adsorption free energy change is likely larger in negative value for *c*-PEG.

### 3.3 Enhancement of the dispersion stability

Subsequently, the dispersion stability of AgNPs_10_/No PEG, AgNPs_10_/HO–PEG_9k_–OH, AgNPs_10_/MeO–PEG_9k_–OMe, AgNPs_10_/HS–PEG_9k_–OMe, and AgNPs_10_/*c*-PEG_9k_ with a PEG concentration of 0.25 wt% under physiological conditions was investigated. On addition of a tenfold-concentrated PBS solution (0.06 mL) to each AgNPs dispersion (0.54 mL) to form the intended physiological conditions (pH 7.4 and a NaCl concentration of 150 mM), there was an immediate color change from yellow to light brown in the case of AgNPs_10_/No PEG and AgNPs_10_/HO–PEG_9k_–OH or to dark brown for AgNPs_10_/MeO–PEG_9k_–OMe (Fig. 2b and ESI Movie 1†), which was followed by precipitation. The dark yellow color of AgNPs_10_/HS–PEG_9k_–OMe remained unchanged. Remarkably, AgNPs_10_/*c*-PEG_9k_ exhibited only a slight color change and nearly retained the initial yellow color even after 1000 min. UV-Vis spectroscopy showed only a minor decrease in the absorption of AgNPs_10_/*c*-PEG_9k_ on addition of the tenfold-concentrated PBS solution and after 1000 min, whereas there was essentially no absorption from AgNPs_10_/No PEG, AgNPs_10_/HO–PEG_9k_–OH, or AgNPs_10_/MeO–PEG_9k_–OMe due to precipitation (Fig. 2c). The relative absorption intensity (Rel. Abs) calculated by dividing the absorption value at *λ*_max_ after 1000 min by that before the addition of PBS was 75% for AgNPs_10_/*c*-PEG_9k_. The presence of NaCl in PBS has been well reviewed as a causative factor of aggregation of metal nanoparticles, but properly performed PEGylation can avoid aggregation.^54^ Thus, *c*-PEG_9k_ protected AgNPs by physisorption to the surface thereby preventing agglomeration in the presence of increased ionic strength. In the meantime, a significant reduction in the UV-Vis absorption spectra of AgNPs_10_/HS–PEG_9k_–OMe was evident after 1000 min (Rel. Abs = 48%), which was likely caused by dissolution of AgNPs in the presence of thiol.^18^

**Fig. 3:**
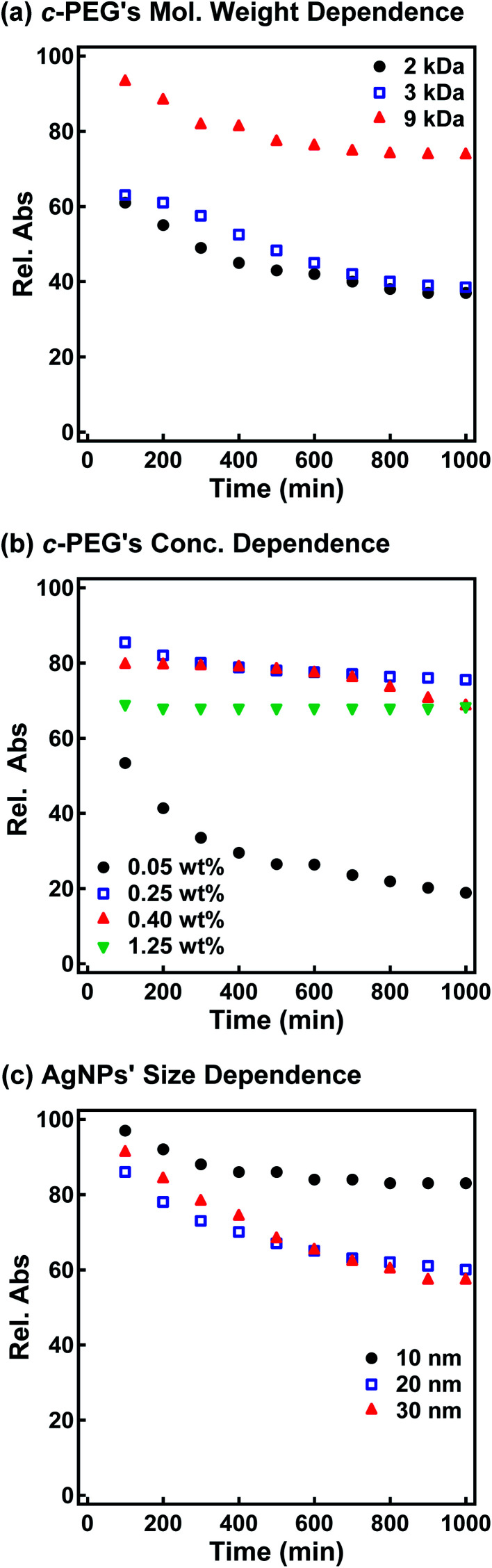
Time course of the relative absorption intensity (Rel. Abs) of AgNPs/*c*-PEG under physiological conditions (PBS with pH 7.4 and a NaCl concentration of 150 mM). Rel. Abs was calculated by dividing the *λ*_max_ absorption value at a given time by that before the addition of PBS. (a) *c*-PEG's molecular weight dependence tested for AgNPs_10_/*c*-PEG_2k_, AgNPs_10_/*c*-PEG_3k_, and AgNPs_10_/*c*-PEG_9k_ with a PEG concentration of 0.25 wt%. (b) *c*-PEG_9k_'s concentration dependence tested for AgNPs_10_/*c*-PEG_9k_ with a PEG concentration of 0.05, 0.25, 0.40, and 1.25 wt%. (c) AgNPs' size dependence tested for AgNPs_10_/*c*-PEG_9k_, AgNPs_20_/*c*-PEG_9k_, and AgNPs_30_/*c*-PEG_9k_ with a PEG concentration of 0.25 wt%.

Subsequently, the stabilization effect depending on the molecular weight of *c*-PEG was investigated. Fig. 3a shows Rel. Abs *versus* time for AgNPs_10_/*c*-PEG_2k_, AgNPs_10_/*c*-PEG_3k_, and AgNPs_10_/*c*-PEG_9k_ in a PBS buffer solution with pH 7.4 and a NaCl concentration of 150 mM. A significant increase in the dispersion stability was observed with an increase in the molecular weight. AgNPs_10_/*c*-PEG_9k_ retained 74% of Rel. Abs after 1000 min, while AgNPs_10_/*c*-PEG_3k_ retained only 39%, and AgNPs_10_/*c*-PEG_2k_ also showed a weak stabilization with Rel. Abs of 37%. Moreover, the concentration of *c*-PEG_9k_ was varied from 0.05 to 1.25 wt% (Fig. 3b). At 0.05 wt%, a continuous decrease in absorbance was seen, and Rel. Abs was 19% after 1000 min. In the meantime, at 0.25 wt% and higher concentrations, there was a much smaller change in time course (Rel. Abs ≥ 76% after 1000 min). At a *c*-PEG concentration of 0.05 wt%, an insufficient amount of *c*-PEG existed in the dispersion as the surface of AgNPs was scarcely covered. At the concentration of 0.25 wt% and higher, the amount of *c*-PEG was satisfactory to form a thick enough layer on the surface of AgNPs, thereby enhancing the dispersion stability. The *c*-PEG layer thickness was likely saturated at 0.25 wt%, and this phenomenon was previously observed for AuNPs.^43^ Furthermore, the stability in relation to the size of AgNPs was also examined. Fig. 3c shows that *c*-PEG_9k_ can stabilize AgNPs_10_, AgNPs_20_, and AgNPs_30_, which have a size of 10, 20 and 30 nm, respectively. However, stability was best conferred on AgNPs_10_/*c*-PEG_9k_ with Rel. Abs of 82% after 1000 min compared to AgNPs_20_/*c*-PEG_9k_ (Rel. Abs = 60%) and AgNPs_30_/*c*-PEG_9k_ (Rel. Abs = 57%). We showed above that the DLS size and *ζ*-potential were dependent on the molecular weight and AgNPs' size (Tables 2 and 3); *c*-PEG_9k_ formed a thicker layer than *c*-PEG_2k_ and *c*-PEG_3k_, on AgNPs_10_ compared to AgNPs_20_ and AgNPs_30_. The dispersion stability against the physiological conditions was in accord with the DLS size and *ζ*-potential; the thicker the PEG layer that forms on AgNPs, the better the dispersion stability is. In accordance with these results, the following experiments were mainly performed with AgNPs_10_/PEG_9k_ with a polymer concentration of 0.25 wt%.

Moreover, divalent ionic salts have been reported to exert stronger dissolution and agglomeration effects on nanoparticles than the monovalent counterparts.^55–57^ On account of stabilization conferred to AgNPs by *c*-PEG_9k_ against PBS with its main constituent as NaCl, we investigated the dispersion stability of AgNPs_10_/No PEG, AgNPs_10_/HO–PEG_9k_–OH, AgNPs_10_/MeO–PEG_9k_–OMe, AgNPs_10_/HS–PEG_9k_–OMe, and AgNPs_10_/*c*-PEG_9k_ with a PEG concentration of 0.25 wt% against a 10 mM CaCl_2_ solution. Similar to the case of the PBS experiment, *c*-PEG conferred stability to AgNPs after 1000 min with Rel. Abs of 78% (Fig. S6†). On the other hand, AgNPs_10_/No PEG, AgNPs_10_/HO–PEG_9k_–OH, and AgNPs_10_/MeO–PEG_9k_–OMe precipitated with Rel. Abs ∼0%, while AgNPs_10_/HS–PEG_9k_–OMe with a shifted and broadened spectrum caused decrease in the absorption (Rel. Abs = 45%). This further proved the strong dispersion stability endowed by *c*-PEG.

**Fig. 4:**
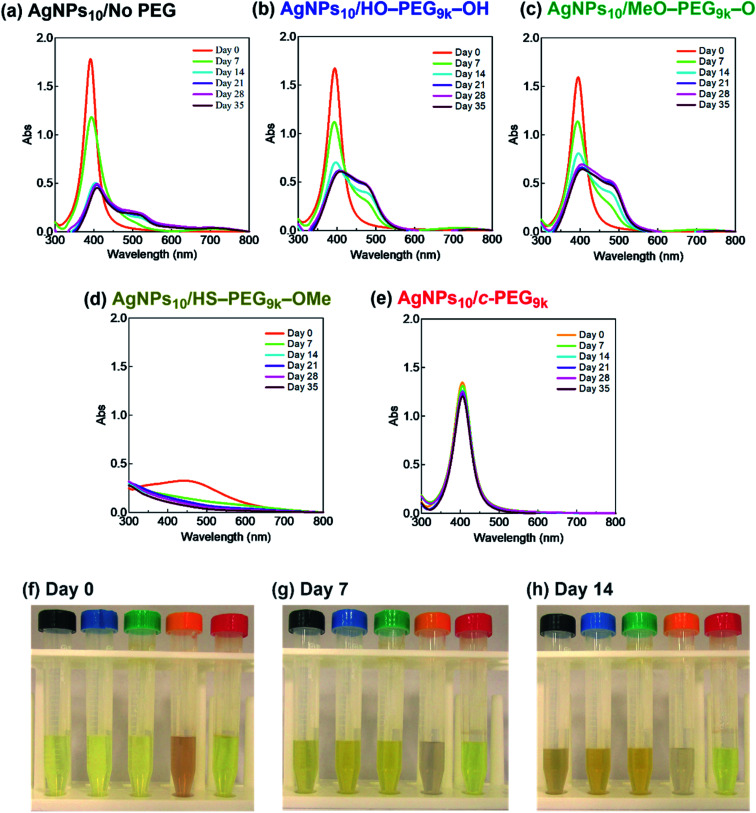
Stability test of AgNPs/PEG against white light of 860–990 lux. UV-Vis spectra of (a) AgNPs_10_/No PEG, (b) AgNPs_10_/HO–PEG_9k_–OH, (c) AgNPs_10_/MeO–PEG_9k_–OMe, (d) AgNPs_10_/HS–PEG_9k_–OMe, and (e) AgNPs_10_/*c*-PEG_9k_ with a PEG concentration of 0.25 wt%. Photographs taken at (f) day 0, (g) day 7, and (h) day 14. In each photograph from left to right: AgNPs_10_/No PEG (black); AgNPs_10_/HO–PEG_9k_–OH (blue); AgNPs_10_/MeO–PEG_9k_–OMe (green); AgNPs_10_/HS–PEG_9k_–OMe (orange); AgNPs_10_/*c*-PEG_9k_ (red).

It was reported that the dissolution, aggregation, and secondary phase precipitation of AgNPs are caused by photoirradiation.^58^ Thus, in production, storage, and applications, light is a well-known limiting factor which causes transformational changes of AgNPs.^59,60^ Also, emphasis is always made in the safety data sheets with respect to light. Thus, we tested the stability endowed by *c*-PEG against photoirradiation. AgNPs_10_/No PEG, AgNPs_10_/HO–PEG_9k_–OH, AgNPs_10_/MeO–PEG_9k_–OMe, AgNPs_10_/HS–PEG_9k_–OMe, and AgNPs_10_/*c*-PEG_9k_ with a PEG concentration of 0.25 wt% were exposed to white light at 860–990 lux (Fig. 4). AgNPs_10_/No PEG (Fig. 4a), AgNPs_10_/HO–PEG_9k_–OH (Fig. 4b), and AgNPs_10_/MeO–PEG_9k_–OMe (Fig. 4c) showed an initial reduction in absorbance at 398 nm, followed by the appearance of a new peak at 550 nm for AgNPs/No PEG and 460–495 nm for AgNPs_10_/HO–PEG_9k_–OH and AgNPs_10_/MeO–PEG_9k_–OMe, which intensified with time. The appearance of AgNPs_10_/No PEG (black-marked tube), AgNPs_10_/HO–PEG_9k_–OH (blue-marked tube), and AgNPs_10_/MeO–PEG_9k_–OMe (green-marked tube) changed from light yellow to deep yellow, which intensified as the days progressed *via* the aggregation of AgNPs (Fig. 4f–h). The strong oscillating dipole–dipole interaction by photoirradiation reportedly causes aggregation.^61^ On the other hand, no obvious change in the UV-Vis spectra in Fig. 4e suggests a superior protection of AgNPs by *c*-PEG_9k_ even after 35 d of exposure to white light, and there was no color change as it remained yellow (red-marked tube in Fig. 4f–h). This was likely because the thick *c*-PEG layer on the surface inhibited the contact of AgNPs and thus prevented the aggregation. In the meantime, AgNPs_10_/HS–PEG_9k_–OMe resulted in reduced absorption of AgNPs immediately after mixing with HS–PEG_9k_–OMe as seen above,^19^ and subsequently gave nearly no absorption from the SPR on day 7 and later (Fig. 4d). The color changed from brownish yellow (orange-marked tube in Fig. 4f) to gray (Fig. 4g and h), which eventually turned to colorless. Moreover, it was also reported that AgNPs coated with PVP cannot withstand photoirradiation, resulting in aggregation.^61^ That is to say, the unique topology of *c*-PEG allows for physisorption to protect AgNPs from degradative reactions taking place at the surface caused by white light exposure.

**Fig. 5:**
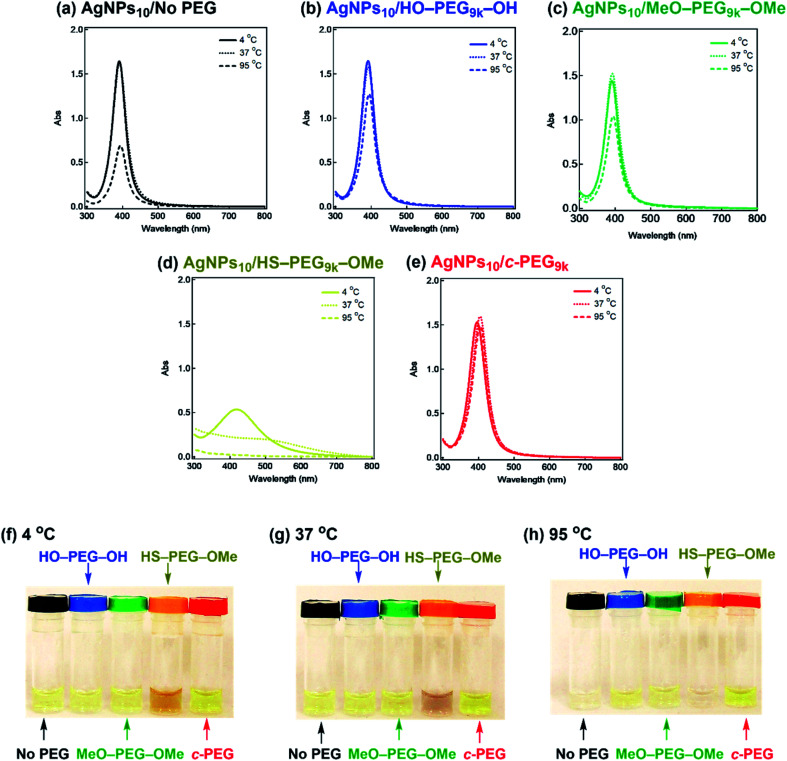
Stability test of AgNPs/PEG against various temperatures. UV-Vis spectra of (a) AgNPs_10_/No PEG, (b) AgNPs_10_/HO–PEG_9k_–OH, (c) AgNPs_10_/MeO–PEG_9k_–OMe, (d) AgNPs_10_/HS–PEG_9k_–OMe, and (e) AgNPs_10_/*c*-PEG_9k_ with a PEG concentration of 0.25 wt% kept at 4, 37, and 95 °C for 4 h. Photographs of the experiments at (f) 4, (g) 37, and (h) 95 °C. In each photograph from left to right: AgNPs_10_/No PEG (black); AgNPs_10_/HO–PEG_9k_–OH (blue); AgNPs_10_/MeO–PEG_9k_–OMe (green); AgNPs_10_/HS–PEG_9k_–OMe (yellow/orange); AgNPs_10_/*c*-PEG_9k_ (red).

It is also known that temperature is another important factor for the aggregation of AgNPs,^61^ and stability against temperature would offer opportunities in various applications such as photothermal therapy.^62,63^ Thus, we further tested the stabilization by *c*-PEG against heating. Keeping AgNPs_10_/No PEG (Fig. 5a), AgNPs_10_/HO–PEG_9k_–OH (Fig. 5b), AgNPs_10_/MeO–PEG_9k_–OMe (Fig. 5c), or AgNPs_10_/*c*-PEG_9k_ (Fig. 5e) at 4 and 37 °C for 4 h gave no change in the absorption spectra. On the other hand, AgNPs_10_/HS–PEG_9k_–OMe (Fig. 5d) showed a red shift and reduced absorption intensity at 4 °C as in the cases of the stability tests against PBS and CaCl_2_.^18^ Further decrease in the absorption spectra of AgNPs_10_/HS–PEG_9k_–OMe was seen when kept at 37 °C with a brownish appearance (orange-marked tube in Fig. 5g). When the temperature was raised to 95 °C, AgNPs_10_/No PEG, AgNPs_10_/HO–PEG_9k_–OH, and AgNPs_10_/MeO–PEG_9k_–OMe after 4 h resulted in Rel. Abs of 42%, 76%, and 72%, respectively, while that of AgNPs_10_/HS–PEG_9k_–OMe was only 5% (Fig. 5a–d). In contrast, AgNPs_10_/*c*-PEG_9k_ showed an insignificant change with a Rel. Abs of 98% under the same conditions (Fig. 5e). After heating at 95 °C for 4 h, AgNPs_10_/No PEG and AgNPs_10_/HS–PEG_9k_–OMe turned colorless, AgNPs_10_/HO–PEG_9k_–OH and AgNPs_10_/MeO–PEG_9k_–OMe gave a slight faint yellow color, whereas AgNPs_10_/*c*-PEG_9k_ remained in the original yellow color (Fig. 5h). Moreover, commercial AgNPs_80_/HS–PEG_5k_–OMe was also heated at 95 °C for 4 h, and there was no conferment of stability to AgNPs as reduction in absorption intensity with disappearance of the yellow color of AgNPs was seen (Fig. S7†). This was in tandem with the above result for AgNPs_10_/HS–PEG_9k_–OMe and further proved the inability of HS–PEG to stabilize AgNPs.

**Fig. 6:**
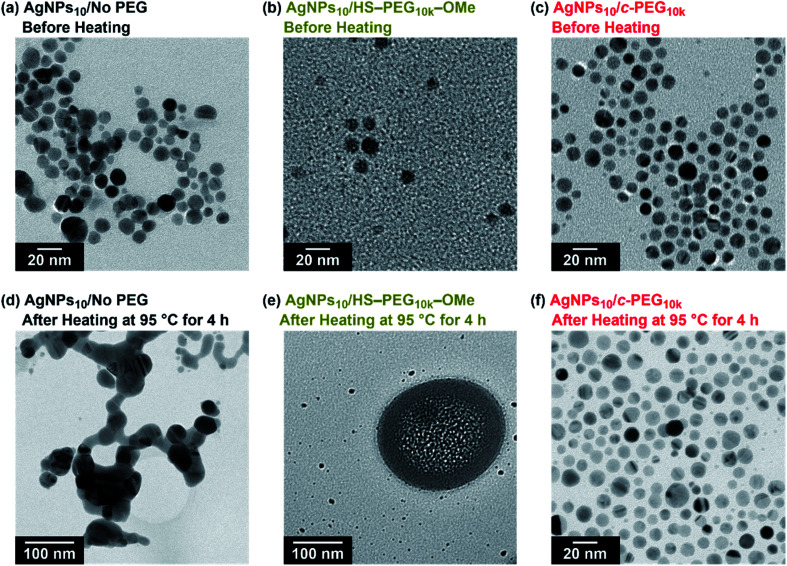
TEM photographs of (a) AgNPs_10_/No PEG (scale bar: 20 nm), (b) AgNPs_10_/HS–PEG_9k_–OMe (scale bar: 20 nm), and (c) AgNPs_10_/*c*-PEG_9k_ (scale bar: 20 nm) before heating. Those of (d) AgNPs_10_/No PEG (scale bar: 100 nm), (e) AgNPs_10_/HS–PEG_9k_–OMe (scale bar: 100 nm), and (f) AgNPs_10_/*c*-PEG_9k_ (scale bar: 20 nm) after heating at 95 °C for 4 h.

TEM measurement of AgNPs_10_/No PEG, AgNPs_10_/HS–PEG_9k_–OMe, and AgNPs_10_/*c*-PEG_9k_ kept at 95 °C for 4 h explained the effect of heating (Fig. 6). TEM photographs of AgNPs_10_/No PEG showed aggregated AgNPs likely caused by the dipole–dipole interaction enhanced at the high temperature (Fig. 6d).^61^ AgNPs_10_/HS–PEG_9k_–OMe after heating drastically also changed its form; particles with reduced size (≤5 nm) along with stain-like objects with a few hundred nanometer in size were observed. The coordination of thiol to the AgNPs surface leads to an Ag_2_S layer.^18,19^ At high temperature, dissociation of Ag_2_S from AgNPs was stimulated to reduce the median particle size shown in Fig. 6e. Moreover, the large stain-like objects were likely Ag_2_S aggregated upon drying. What needs to be emphasized here is that AgNPs_10_/*c*-PEG_9k_ was intact after heating with no significant change in size, and the particles were still well dispersed (Fig. 6f). Because the nanoparticles were separated from each other by the *c*-PEG layer formed on the surface, aggregation of AgNPs did not take place to retain the original size and shape. Concerning this, the nanoparticles of AgNPs_10_/*c*-PEG_9k_ observed in Fig. 6c and f were separated from each other likely by the *c*-PEG layer, compared to those of AgNPs/No PEG in contact with each other as shown in Fig. 6a. These experiments demonstrate how AgNPs/No PEG and AgNPs/HS–PEG–OMe are degraded at high temperature, and *c*-PEG serves as an effective protection for AgNPs.

### 3.4 Biological applications

**Fig. 7:**
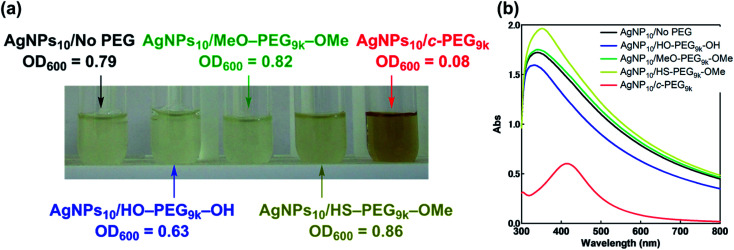
(a) Photograph and (b) UV-Vis spectra of the antimicrobial efficacy test against *E. coli* after 24 h of incubation with AgNPs_10_/No PEG, AgNPs_10_/HO–PEG_9k_–OH, AgNPs_10_/MeO–PEG_9k_–OMe, AgNPs_10_/HS–PEG_9k_–OMe, and AgNPs_10_/*c*-PEG_9k_. UV-Vis spectra of the incubated specimens subtracted by that of the medium are shown.

Antimicrobial activity is inherent and one of the most important properties of AgNPs, and that against Gram-negative *Escherichia coli* (JM 109) in a Muller Hinton Broth (MHB) medium was evaluated. Thus, AgNPs_10_/No PEG, AgNPs_10_/HO–PEG_9k_–OH, and AgNPs_10_/MeO–PEG_9k_–OMe, AgNPs_10_/HS–PEG_9k_–OMe, and AgNPs_10_/*c*-PEG in PBS (pH 7.4, NaCl 150 mM) were added to an *E. coli*-containing medium. Upon addition of AgNPs_10_/*c*-PEG to *E. coli*, immediate change in the color to brownish was observed, while the other mixtures did not cause the change. After 24 h of incubation, the *E. coli*-containing medium with AgNPs_10_/No PEG, AgNPs_10_/HO–PEG_9k_–OH, and AgNPs_10_/MeO–PEG_9k_–OMe became cloudy, suggesting the growth of *E. coli* (Fig. 7a). For AgNPs_10_/HS–PEG_9k_–OMe, a gradual disappearance of the transparent yellow color of AgNPs to form a cloudy solution was evident, which also suggests loss of antimicrobial activity. However, the medium with AgNPs_10_/*c*-PEG_9k_ was transparent and remained brownish in color even after 24 h, showing inhibited growth of *E. coli*. Furthermore, UV-Vis spectra were recorded to show transparency of the AgNPs_10_/*c*-PEG_9k_ specimen with a clearly observable SPR absorption peak, while the other specimens were turbid with a considerably increased baseline through scattering by grown *E. coli* (Fig. 7b). The growth of *E. coli* was quantified by optical density at 600 nm (OD_600_): AgNPs_10_/No PEG, 0.79; AgNPs_10_/HO–PEG_9k_–OH, 0.63; AgNPs_10_/MeO–PEG_9k_–OMe, 0.82; AgNPs_10_/HS–PEG_9k_–OMe, 0.86; AgNPs_10_/*c*-PEG_9k_, 0.08. This revealed that the antimicrobial efficacy was preserved in AgNPs_10_/*c*-PEG_9k_ but lost in all other specimens. Because HO–PEG_9k_–OH and MeO–PEG_9k_–OMe could not maintain the dispersibility of AgNPs in PBS, the antimicrobial potency was quenched before the addition to *E. coli*. In the case of AgNPs_10_/HS–PEG_9k_–OMe, no precipitate formed, but the sulfidation of AgNPs nullified the antimicrobial efficacy. In contrast, physisorption of *c*-PEG exhibited an improved dispersion stability of AgNPs and evidently retained the antimicrobial efficacy.

Following the various dispersion stability and antimicrobial activity experiments, further evaluation of the biological properties of AgNPs/*c*-PEG *via* cytotoxicity and scratch assay experiments using HeLa cells were performed. Fig. S8† shows the result of cytotoxicity of AgNPs_10_/No PEG, AgNPs_10_/HO–PEG_9k_–OH, AgNPs_10_/MeO–PEG_9k_–OMe, AgNPs_10_/HS–PEG_9k_–OMe, and AgNPs_10_/*c*-PEG_9k_ after incubation with HeLa cells in a DMEM medium for 24 h. AgNPs_10_/*c*-PEG_9k_ had the lowest cell viability of 79% which was statistically significant (*p* < 0.05) compared to AgNPs_10_/No PEG with a viability of 85% with sextuplicate experiments. This suggests that *c*-PEG helps in the dispersion of AgNPs in the medium and preserves the cytotoxicity. On the other hand, no cytotoxicity was seen in AgNPs_10_/HO–PEG_9k_–OH, AgNPs_10_/MeO–PEG_9k_–OMe, and AgNPs_10_/HS–PEG_9k_–OMe likely due to precipitation under the conditions.

In addition, migration and recovery of HeLa cells were evaluated by a cell scratch assay. A confluent monolayer was scratched on AgNPs_10_/No PEG, AgNPs_10_/HO–PEG_9k_–OH, AgNPs_10_/MeO–PEG_9k_–OMe, AgNPs_10_/HS–PEG_9k_–OMe, and AgNPs_10_/*c*-PEG_9k_ after 2 h of incubation (Fig. S9†). The scratches in AgNPs_10_/No PEG, AgNPs_10_/HO–PEG_9k_–OH, AgNPs_10_/MeO–PEG_9k_–OMe, and AgNPs_10_/HS–PEG_9k_–OMe specimens were recovered to some extent after 22 h. However, most of the HeLa cells in AgNPs_10_/*c*-PEG_9k_ were stripped off from the plates upon scratching, and basically no recovery was observed. This could be explained by AgNPs_10_/*c*-PEG_9k_ leading to cell death in the large area, thereby inhibiting adhesion of the cells. These results further confirmed the cytotoxicity through the enhanced dispersion stability of AgNPs by *c*-PEG.

## 4. Conclusions

Our research has shown the first steady PEGylation method for AgNPs conferred by physisorption of *c*-PEG, which cannot be attained with HS–PEG–OMe due to the formation of silver sulfide. Physisorption of *c*-PEG provided outstanding dispersion stability to AgNPs, against physiological conditions, white light, and high temperature, whereas HO–PEG–OH or MeO–PEG–OMe did not provide such dispersion stability. This method further exhibited persistent antimicrobial activity and cytotoxicity, which are two of the most important properties of AgNPs. Coupled with the excellent biocompatibility of PEG and the simple physisorption method, *c*-PEG would pave the way and broaden the uses of AgNPs especially in the biological and medicinal fields. Moreover, as we previously proved that the physisorption of *c*-PEG can also enhance the dispersion stability of AuNPs,^43^ the present method has great potential for application in a wide variety of metal nanoparticles.

## Conflicts of interest

The authors declare no competing interest.

## Supplementary Material

NA-004-D1NA00720C-s001

NA-004-D1NA00720C-s002
