# Supplementary material for: PEGylation of silver nanoparticles by physisorption of cyclic poly(ethylene glycol) for enhanced dispersion stability, antimicrobial activity, and cytotoxicity
Source: Nanoscale Adv. 2021 Nov 12;4(2):532–45. doi: 10.1039/d1na00720c (PMC9417676; doi:10.1039/d1na00720c)
Supplement: NA-004-D1NA00720C-s002 [file NA-004-D1NA00720C-s002.pdf]

Electronic Supplementary Material (ESI) for Nanoscale Advances.
This journal is © The Royal Society of Chemistry 2021

Electronic supplementary information

PEGylation of silver nanoparticles by physisorption of cyclic poly(ethylene glycol) for enhanced dispersion stability, antimicrobial activity, and cytotoxicity

Oziri Onyinyechukwu J.,[a] Yubo Wang,[a] Tomohisa Watanabe,[a] Shuya Uno,[a] Masatoshi Maeki,[b] Manabu Tokeshi,[b] Takuya Isono,[b] Kenji Tajima,[b] Toshifumi Satoh,[b] Shin-ichiro Sato,[b] Yutaka Miura,[c] and Takuya Yamamoto[*b]

[a]*Graduate School of Chemical Sciences and Engineering, Hokkaido University, Sapporo, Hokkaido 060–8628, Japan.*

[b]*Division of Applied Chemistry, Faculty of Engineering, Hokkaido University, Sapporo, Hokkaido 060–8628, Japan. E-mail: yamamoto.t@eng.hokudai.ac.jp*

[c]*Laboratory for Chemistry and Life Science, Institute of Innovative Research, Tokyo Institute of Technology, 4259 Nagatsutacho, Midori-ku, Yokohama, Kanagawa, 226–8503, Japan.*

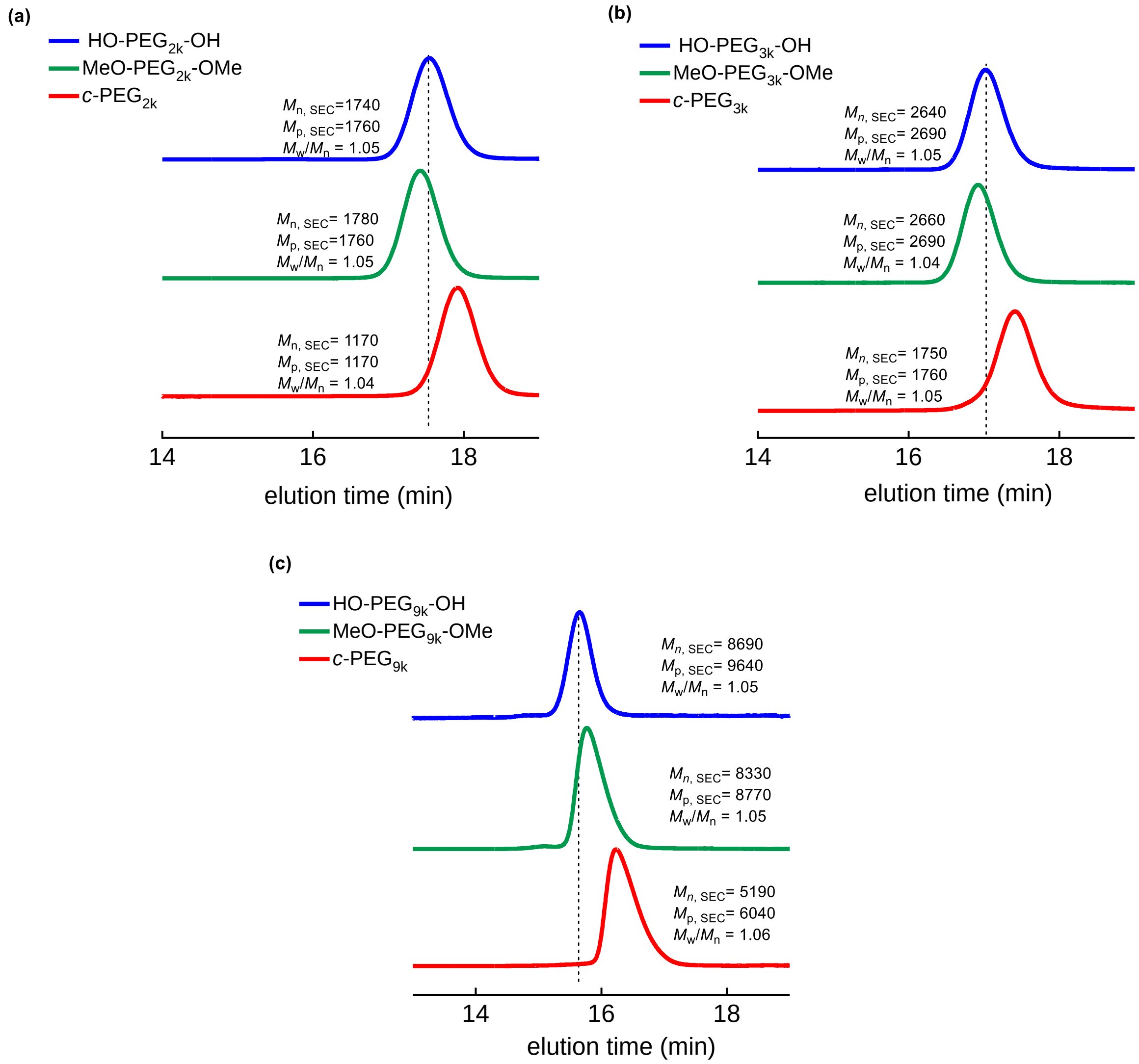

Fig. S1 SEC traces of HO–PEG–OH (blue), MeO–PEG–OMe (green) and *c*-PEG (red) with a molecular weight of (a) 2, (b) 3, and (c) 9 kDa.

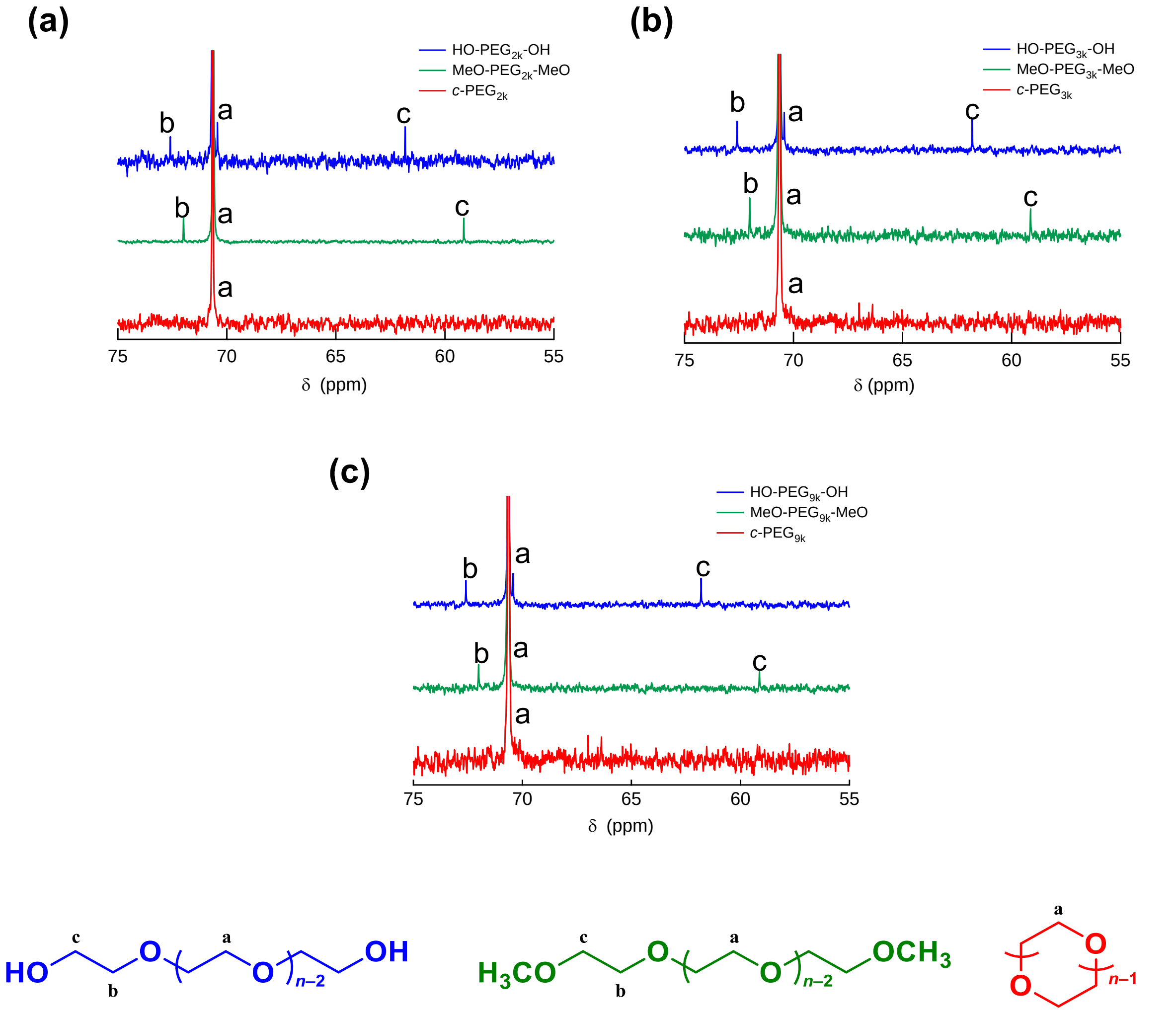

Fig. S2 ^{13}C NMR spectra of HO–PEG–OH (blue), MeO–PEG–OMe (green), and *c*-PEG (red) with a molecular weight of (a) 2, (b) 3, and (c) 9 kDa.

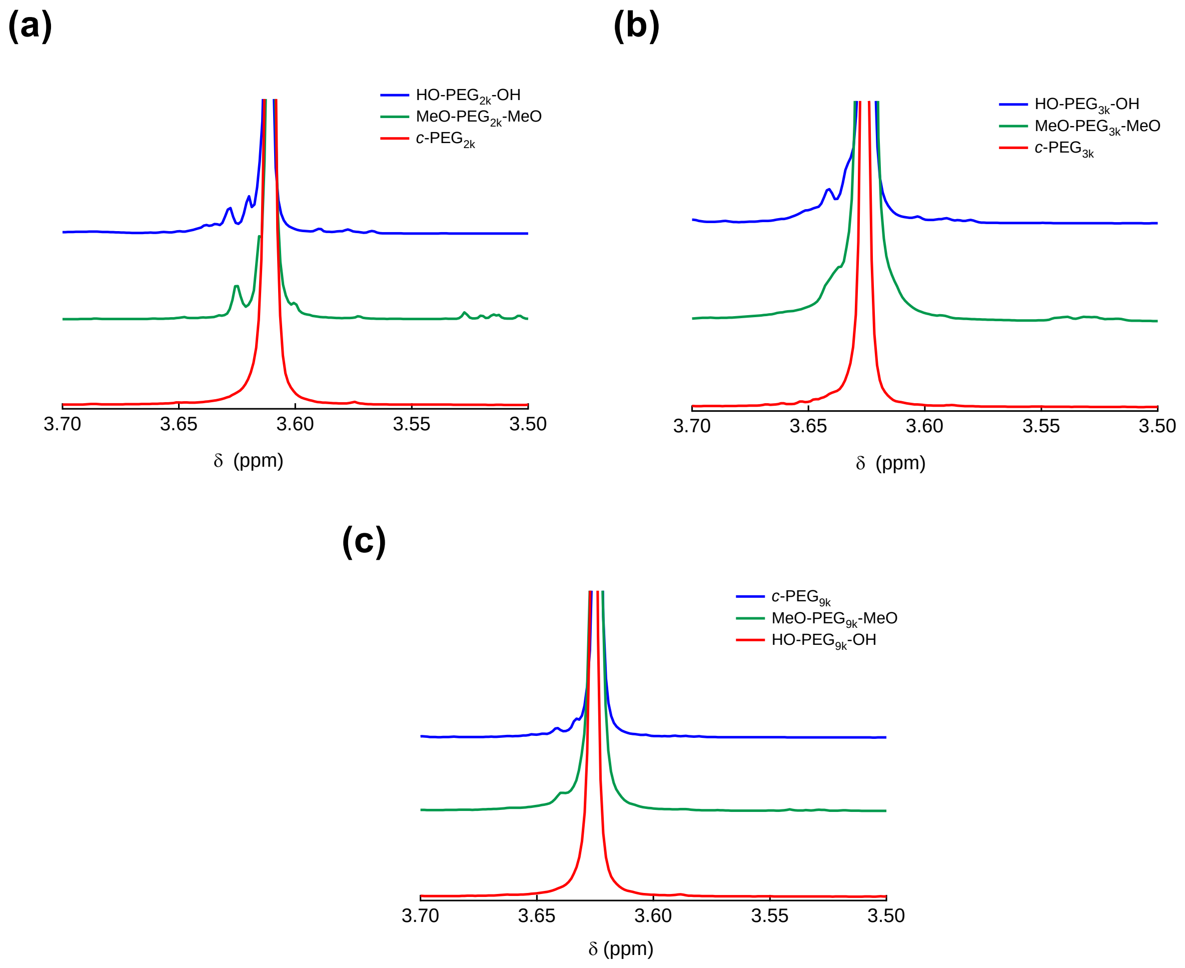

Fig. S3 ^{1}H NMR spectra of HO–PEG–OH (blue), MeO–PEG–OMe (green), and *c*-PEG (red) with a molecular weight of (a) 2, (b) 3, and (c) 9 kDa.

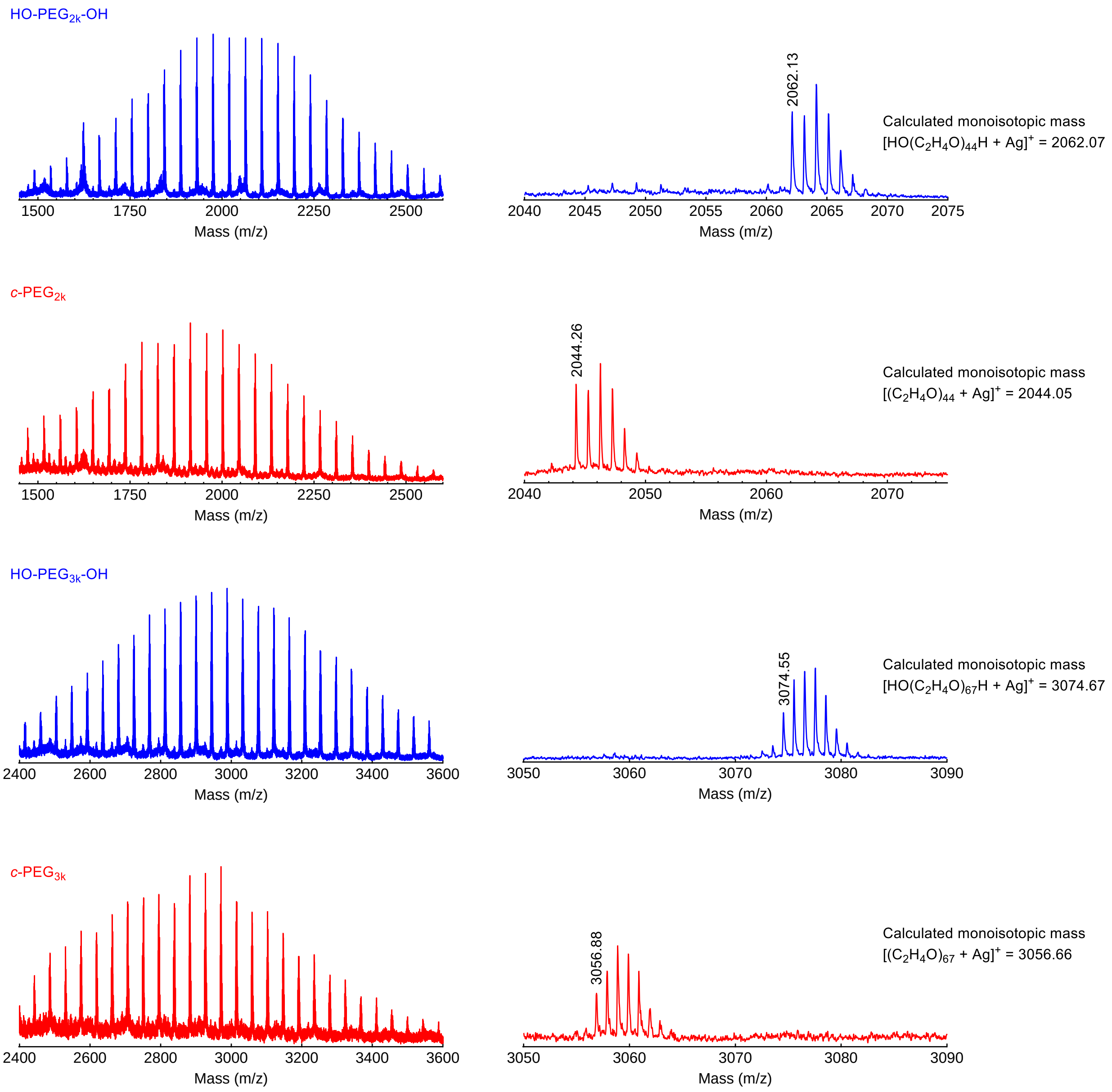

Fig. S4 MALDI-TOF mass spectra of HO–PEG–OH (blue) and *c*-PEG (red) with a molecular weight of (a) 2 and (b) 3kDa.

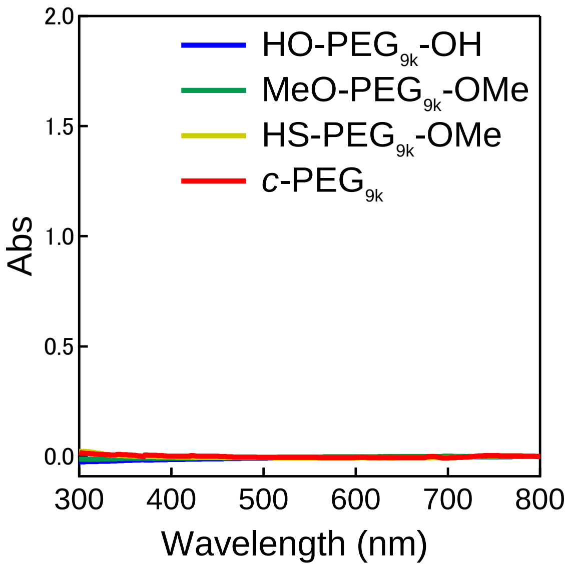

Fig. S5 UV–Vis spectra of HO–PEG$_{9k}$–OH (blue), MeO–PEG$_{9k}$–OMe (green), HS–PEG$_{9k}$–OMe (yellow), and *c*-PEG$_{9k}$ (red).

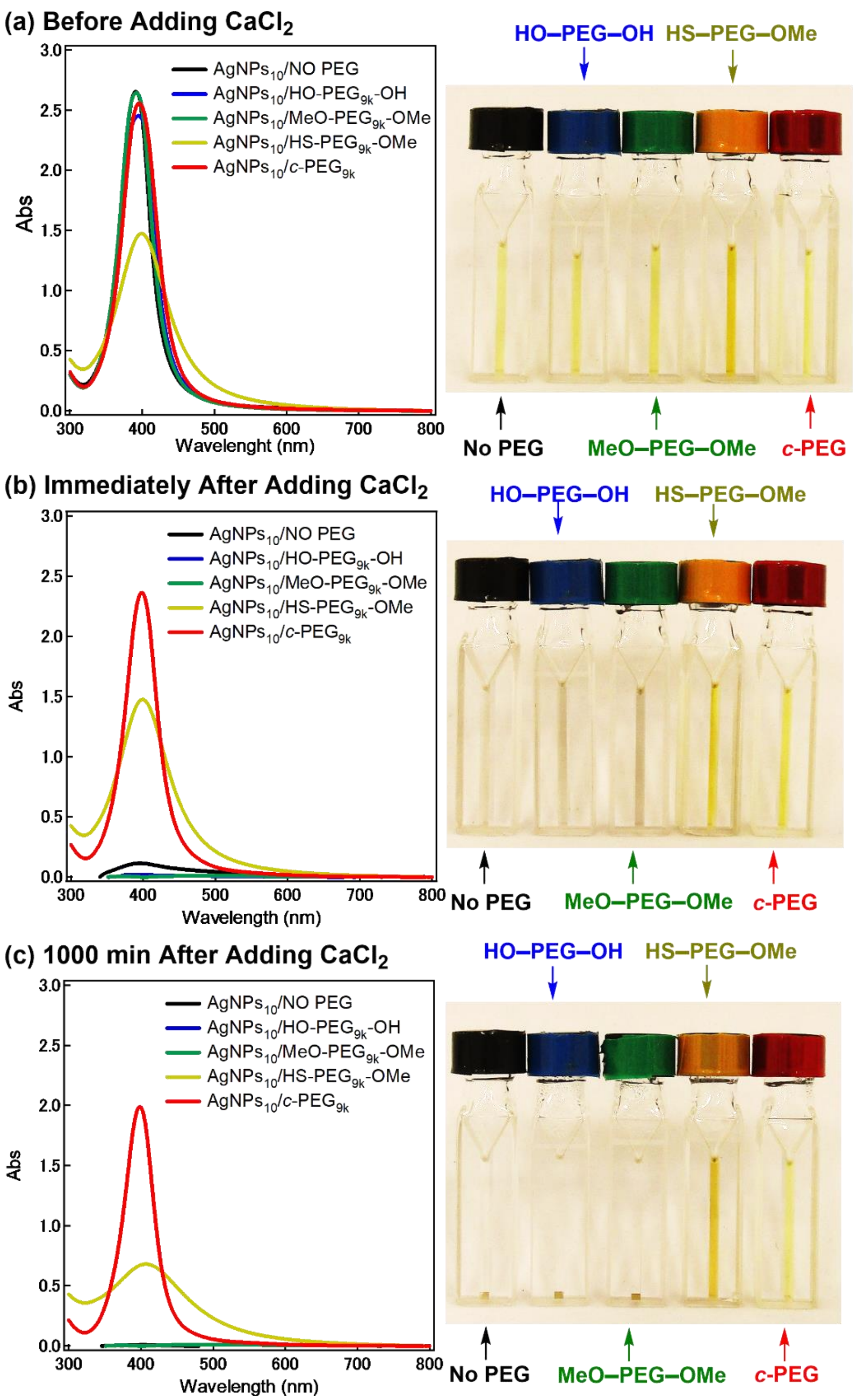

Fig. S6 Stability test of AgNPs/PEG against $CaCl_2$. UV–Vis spectra and photographs of $AgNPs_{10}$/No PEG (black), $AgNPs_{10}$/HO–PEG_{9k}–OH (blue), $AgNPs_{10}$/MeO–PEG_{9k}–OMe (green), $AgNPs_{10}$/HS–PEG_{9k}–OMe (yellow/orange), and $AgNPs_{10}$/*c*-PEG_{9k} (red) with a PEG concentration of 0.25 wt% (a) before (b) immediately after and (c) 1000 min after the addition of a concentrated $CaCl_2$ solution. The resulting dispersions had 10 mM of $CaCl_2$.

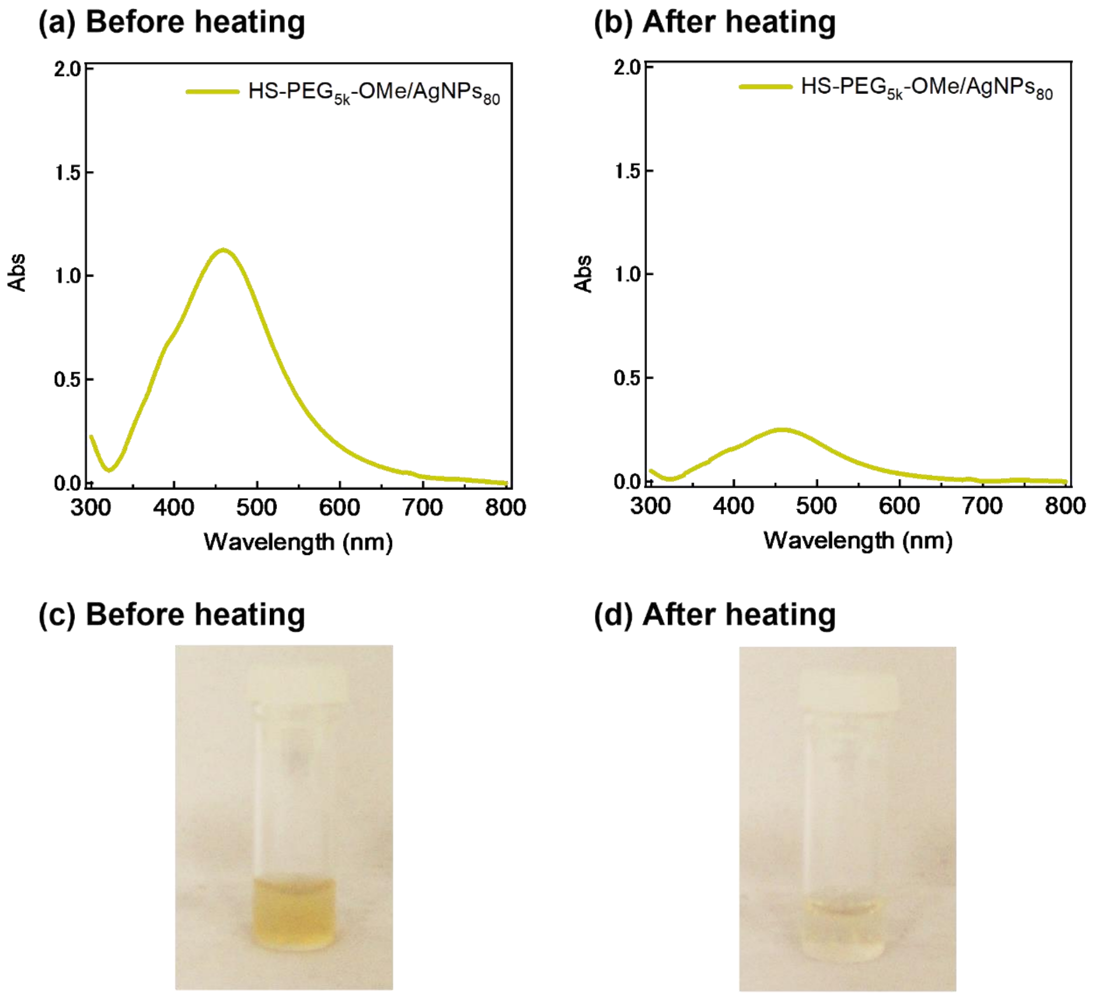

Fig. S7 Stability test of commercial $AgNPs_{80}$/HS–PEG_{5k}–OMe against heating. UV–Vis spectra of $AgNPs_{80}$/HS–PEG_{5k}–OMe (a) before heating and (b) after heating at 95 °C for 4 h. Photographs of $AgNPs_{80}$/HS–PEG_{5k}–OMe (c) before heating and (d) after heating at 95 °C for 4 h.

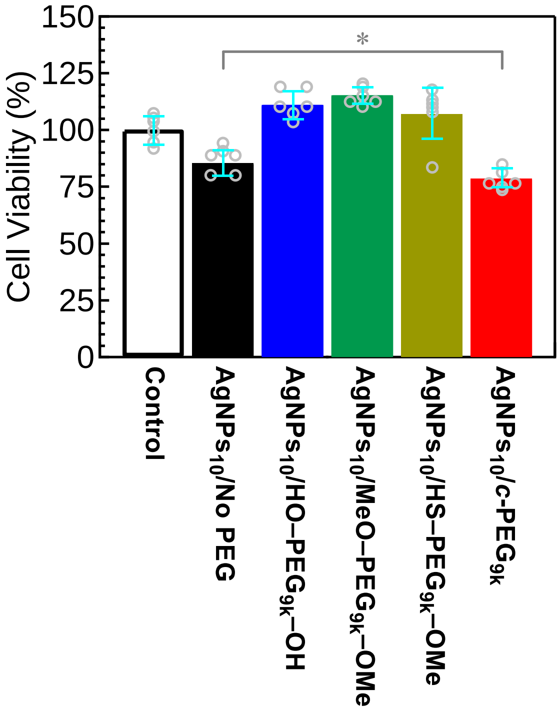

Fig. S8 Cell viability for $AgNPs_{10}$/No PEG, $AgNPs_{10}$/HO–PEG_{9k}–OH, $AgNPs_{10}$/MeO–PEG_{9k}–OMe, $AgNPs_{10}$/HS–PEG_{9k}–OMe, and $AgNPs_{10}$/*c*-PEG_{9k}. Data represent mean ± s.e. from measurements of six wells. *$p < 0.05$.

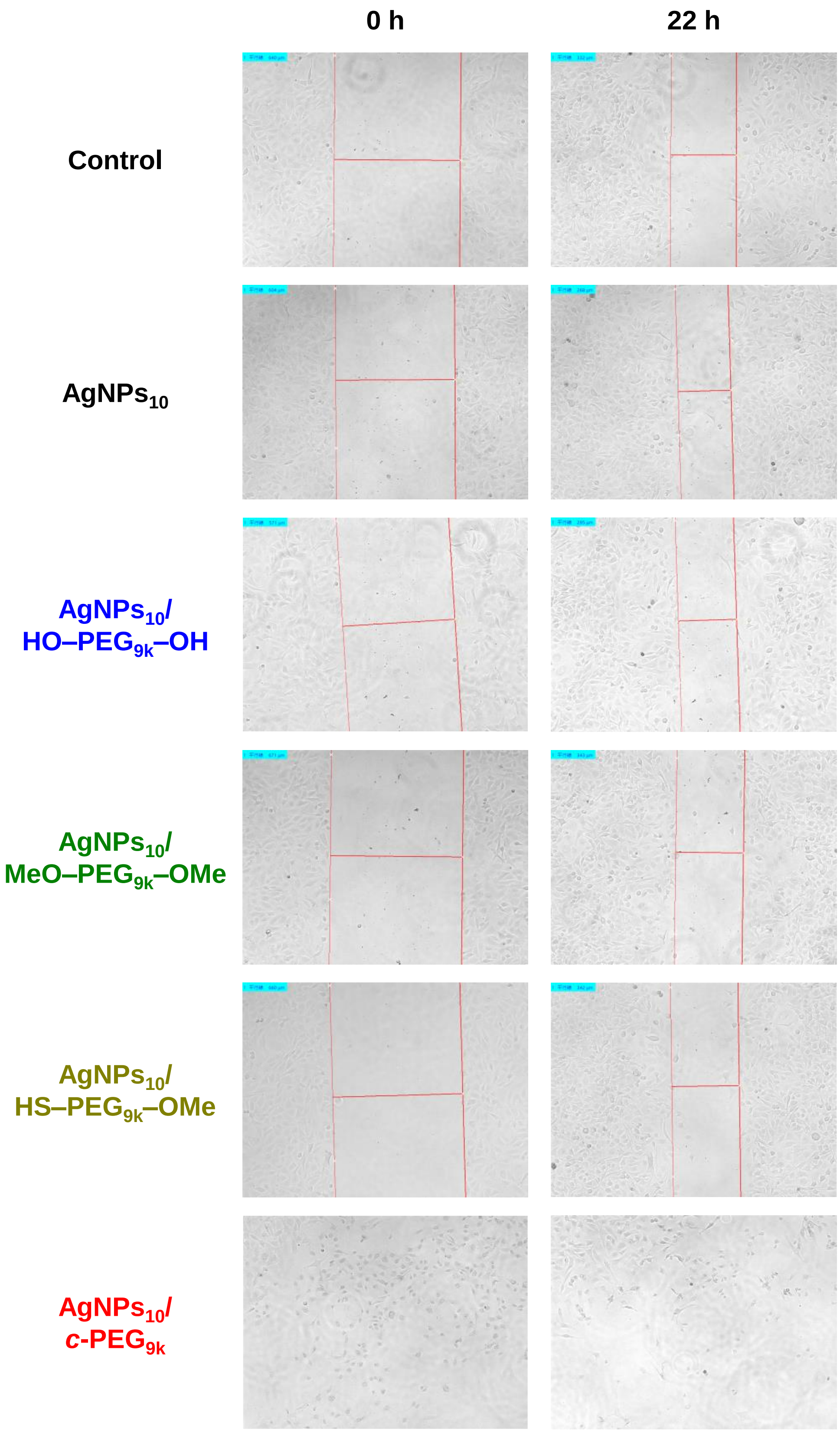

Fig. S9 Scratch assay test for $AgNPs_{10}$/No PEG, $AgNPs_{10}$/HO–PEG_{9k}–OH, $AgNPs_{10}$/MeO–PEG_{9k}–OMe, $AgNPs_{10}$/HS–PEG_{9k}–OMe, and $AgNPs_{10}$/*c*-PEG_{9k}. The pictures were taken at 0 and 22 h after scratching. Migration of the cells into the scratched area was observed in all the specimens except for $AgNPs_{10}$/*c*-PEG_{9k}. Most of the cells in $AgNPs_{10}$/*c*-PEG_{9k} were stripped from the plate upon scratching.
